# The “Tianyu” Formulation Alleviates Rheumatoid Arthritis by Modulating the NLRP3/Caspase‐1/GSDMD‐Mediated Pyroptosis Pathway

**DOI:** 10.1096/fj.202504611R

**Published:** 2026-04-25

**Authors:** Yueya Zhu, Xinying Cai, Qiuhan Zheng, Chunqiu Fang, Zhi Pan, Yinghang Wang

**Affiliations:** ^1^ Jilin Ginseng Academy Changchun University of Chinese Medicine Changchun China; ^2^ College of Traditional Chinese Medicine Changchun University of Chinese Medicine Changchun China; ^3^ Department of Rheumatology and Immunology The Affiliated Hospital of Changchun University of Chinese Medicine Changchun China

**Keywords:** NLRP3 inflammasome, pyroptosis, RA‐HFLS, rheumatoid arthritis, Tianyu formulation

## Abstract

This research focuses on exploring the modulation of pyroptosis and its underlying mechanisms via the “Tianyu” formulation (TY), a traditional Chinese medicine, in human rheumatoid arthritis fibroblast‐like synovial cells (RA‐HFLS) and a collagen‐induced arthritis (CIA) rat model. In vitro, RA‐HFLS cells were induced with tumor necrosis factor α (TNF‐α). Cell viability, proliferation, migration, and invasion were assessed using CCK‐8, EdU, and Transwell assays, respectively. Pyroptosis was evaluated by flow cytometry, acridine orange/ethidium bromide (AO/EB) staining, and by measuring the release of lactate dehydrogenase (LDH) and inflammatory factors (interleukin [IL]‐1β, IL‐18, TNF‐α) in the cell culture supernatants. The expression of pyroptosis‐related mRNAs and proteins was examined by RT‐qPCR, immunofluorescence, and Western blotting. For in vivo analysis, a CIA rat model was established. Joint swelling was evaluated using arthritis scores and paw volume measurements, while histopathological changes in the joint tissues were examined by hematoxylin and eosin (HE) staining and Safranin O–Fast Green staining. Serum levels of autoantibodies (anti‐CCP, RF), LDH, and inflammatory cytokines (IL‐1β, IL‐18, TNF‐α) were quantified concurrently. Furthermore, the expression of pyroptosis‐related molecules in joint tissues was determined using RT‐qPCR, immunohistochemistry, and Western blot analysis. The major components of TY were identified by UHPLC‐Q Exactive HFX. Subsequently, molecular docking simulations were performed to evaluate the binding affinities of the main bioactive compounds to the NLRP3, Caspase‐1, and GSDMD proteins. In vitro experiments showed that TY significantly reduced the viability, proliferation, migration, and invasion abilities, as well as the LDH levels of TNF‐α‐induced RA‐HFLS cells, and decreased the levels of IL‐1β, IL‐18, and TNF‐α in the supernatant. Additionally, TY downregulated the expression of key proteins involved in pyroptosis mediated by the NOD‐like receptor family pyrin domain containing 3 (NLRP3) inflammasome. In the CIA rat model, TY treatment alleviated arthritis symptoms, reduced paw swelling and bone erosion, improved joint pathology, decreased serum levels of IL‐1β, IL‐18, and TNF‐α, and suppressed the expression of key proteins involved in the NLRP3 inflammasome‐mediated pyroptosis pathway in synovial tissue. A total of 95 compounds belonging to 16 classes were identified from TY in both positive and negative ion scanning modes. These included 42 phenylpropanoid and polyphenolic compounds, as well as 12 organic oxides. Molecular docking showed that all five major compounds (Apigenin, Isorhamnetin, Kaempferol, Quercetin, and Salidroside) bound well to NLRP3, Caspase‐1, and GSDMD, with all binding energies below −5 kcal/mol. Among them, Quercetin exhibited the highest affinity for GSDMD (−9.1 kcal/mol). By regulating the NLRP3/Caspase‐1/GSDMD pathway, TY alleviates joint inflammation in CIA rats and reduces pyroptosis in RA‐HFLS cells.

## Graphical abstract

Proposed signaling pathway by which TY modulates NLRP3/Caspase‐1/GSDMD‐mediated pyroptosis.

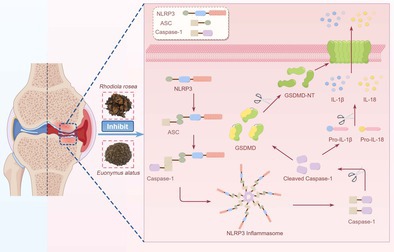

## 1 Introduction

Rheumatoid arthritis (RA) is a long‐term systemic condition that involves chronic inflammation, primarily affecting the synovial membranes and leading to the formation of vascular villi, with clinical manifestations primarily featuring symmetrical polyarticular swelling and pain [1, 2]. As the disease progresses, cartilage and bone joints are progressively eroded by inflammation, leading to joint deformities and functional loss that severely impact patients' quality of life [3].

In healthy individuals, the synovial membrane comprises superficial layers of fibroblasts and macrophages, with underlying layers containing fibroblasts, macrophages, and adipocytes, while the superficial macrophages form a physical barrier through tight junctions, protecting the joint cavity from immune cell infiltration [4]. However, studies reveal that in chronic synovitis of RA, the structural integrity of the synovial surface layer is disrupted, leading to the loss of the tight junction barrier and allowing immune cells, including fibroblasts, to infiltrate the joint cavity [5]. Activated fibroblasts and underlying fibroblasts trigger inflammatory responses, ultimately causing synovial inflammation and tissue damage within the joint cavity [6]. Activation of inflammasomes and subsequent pyroptosis can trigger an inflammatory response and recruit myeloid cells, a mechanism that has been validated in various chronic inflammatory diseases [7], indicating its potential involvement in RA immunopathology. The inflammasome is a multiprotein signaling complex that responds to danger signals in the cytoplasm and activates Caspase‐1, thereby inducing pyroptosis [8]. Pyroptosis is an inflammatory form of programmed cell death that triggers cell rupture through activation of Gasdermin (GSDM) proteins, releasing inflammatory mediators such as Interleukin‐1β (IL‐1β) and Interleukin‐18 (IL‐18) [9]. In the canonical activation pathway, NOD‐like receptor family pyrin domain containing 3 (NLRP3) inflammasomes are activated by pathogen‐associated molecular patterns (PAMPs) or damage‐associated molecular patterns (DAMPs) [10]. Subsequently, Caspase‐1 cleaves the GSDMD protein, releasing its N‐terminal domain (GSDMD‐NT), leading to pore formation in the cell membrane, triggering cell swelling and rupture, releasing inflammatory mediators and exacerbating the inflammatory response [11, 12]. As pyroptosis persists, the inflammatory response intensifies, ultimately forming a vicious cycle in the immunopathology of RA, driving chronic joint inflammation and tissue damage, and eventually leading to the loss of joint function. Therefore, intervention strategies targeting pyroptosis may provide a new therapeutic direction for breaking this vicious cycle.

Currently, commonly used clinical medications for RA, including non‐steroidal anti‐inflammatory drugs (NSAIDs), glucocorticoids (GCs), and disease‐modifying antirheumatic drugs (DMARDs), can effectively alleviate symptoms [13, 14]. However, these medications are associated with issues such as cardiovascular adverse events (NSAIDs) [15, 16], metabolic disorders (GCs) [17], and organ toxicity (DMARDs) [18]. In contrast, traditional Chinese medicine (TCM) formulations, with their multi‐target regulatory effects and relatively good safety profile, have been shown to improve RA conditions and contribute to immune homeostasis [19, 20]. For example, studies have shown [21] that Pheretima vulgaris effectively reduce synovial CD4^+^ T cell infiltration and lower inflammatory cytokine levels by inhibiting the CXCL10/CXCR3 chemotactic signaling pathway, thereby alleviating joint inflammation and damage. Despite the therapeutic efficacy of TCM in RA, its role in the pyroptosis mechanism remains underexplored. Therefore, investigating the potential of TCM in regulating pyroptosis offers important insights for the development of new drugs.

The Tianyu formulation (TY) consists of two Chinese herbal ingredients, *Rhodiola crenulata* and *Euonymus alatus* . *Rhodiola crenulata* has been widely used in TCM to treat inflammatory diseases, alleviate joint pain, and modulate immunity [22]. Salidroside, the primary active component in *Rhodiola crenulata*, is capable of reshaping the Th17/Treg immune balance by regulating the STAT3/HIF‐1α/RORγt signaling axis. This mechanism reduces Th17 cell differentiation and upregulates Treg function, which consequently alleviates RA joint inflammation and cartilage destruction [23]. *Euonymus alatus* contains various bioactive components and exhibits multiple pharmacological effects, including antitumor, antihypertensive, antidiabetic, and immunomodulatory activities [24]. Research indicates that *Euonymus alatus* is capable of alleviating inflammatory bone erosion in RA by inhibiting the RANKL signaling pathway, thereby reducing osteoclast differentiation and bone resorption activity [25]. Preliminary laboratory studies have investigated the application of TY preparations in RA treatment [26, 27]. However, the specific mechanisms underlying the action of the TCM compound TY, particularly in relation to pyroptosis and its active ingredients, have not been systematically studied.

Therefore, the purpose of this study is to investigate the mechanism by which TY regulates the NLRP3/Caspase‐1/GSDMD signaling pathway in the treatment of RA, utilizing ultra‐high‐performance liquid chromatography coupled with high‐resolution mass spectrometry (UHPLC‐Q Exactive HFX), RA fibroblast‐like synovial (RA‐HFLS) cells, and collagen‐induced arthritis (CIA) rat models for multi‐dimensional validation. Furthermore, this investigation seeks to explore the correlation between its active ingredients and pharmacodynamic effects, thereby providing novel mechanistic insights and therapeutic strategies for the treatment of RA using TCM compounds.

## 2 Materials and Methods

### 2.1 Preparation of TY

The TY consists of 10 g *Rhodiola rosea* and 10 g *Euonymus alatus* . All herbs were sourced from Jilin Provincial Beiyao TCM Pharmaceutical Group (Jilin Province, China). Using the body surface area conversion between humans and rats, as outlined in Pharmacological Experimental Methods, the equivalent dose for rats was determined to be 2.1 g·kg^−1^, classified as the medium dose [27]. The low dose was defined at 1.05 g·kg^−1^ and the high dose at 4.2 g·kg^−1^ [27]. The dose for Tripterygium glycosides tablets (191204, Shanghai Fudan Fuhua Pharmaceutical) was determined to be 9.45 mg·kg^−1^ using the same methodology. Specified amounts of TY are weighed, prepared into high, medium, and low dosages, soaked in distilled water for 1 h, and then boiled twice for 1.5 h each in 10 times the volume of distilled water. For subsequent use, concentrate the two filtrate solutions and store them at 4°C. For the suspension preparation, Tripterygium glycoside tablets were mixed with distilled water and stored at 4°C for later use.

### 2.2 Cell Induction and Therapy

#### 2.2.1 Preparation of TY Serum

After 1 week of acclimatization, 30 female Wistar rats (weighing 180–200 g) were randomly assigned to the control group (blank control group and model group, 5 rats/group, 10 rats in total) and the treatment group (Tripterygium glycosides group and TY high, medium, low‐dose groups, 5 rats/group, 20 rats in total). All animal experiments were conducted in accordance with the policies of the US Public Health Service on the use of laboratory animals and were approved by the Ethics Committee of Changchun University of Chinese Medicine (Approval No. 2024842). The control group was administered distilled water via gastric lavage, while the remaining groups were administered gastric lavage at the doses described in Section 2.1 for three consecutive days. Rats in all groups received an intraperitoneal injection of 20% urethane to induce anesthesia 2 h after the last treatment on Day 3. Blood was then drawn from the abdominal aorta. Serum was extracted by centrifugation after blood was allowed to stand at room temperature for 2 h. After 30 min of inactivation in a water bath at 56°C, the serum was kept at −20°C for future use.

#### 2.2.2 Cell Culture, Induction, and Therapy

For in vitro experimental validation, RA‐HFLS cells (Guangzhou Genio, Guangzhou, China) were utilized. RA‐HFLS cells were cultured in RPMI 1640 medium (C11875500BT, Gibco, USA), supplemented with 10% fetal bovine serum (FBS, FSP500, Excell, Suzhou, China) and 1% penicillin–streptomycin solution (C3420‐0100, Vivacell, Shanghai, China). Cells were maintained in an incubator set to 5% CO2 at 37°C. After stimulation with 10 ng/mL tumor necrosis factor alpha (TNF‐α, 300‐01A, ProTech, USA) for 24 h [28], the RA‐HFLS cells were divided into the following groups: control (Con), TNF‐α, TG, TY‐H, TY‐M, and TY‐L. Cells were treated with 10% drug‐containing sera for 24 h, after which further tests were conducted.

### 2.3 Assay for CCK‐8 Cell Viability and EdU Cell Proliferation

The Cell Counting Kit‐8 (CCK‐8) assay (K1018, APEXBIO, USA) was used to measure cell proliferation for cells treated with TY serum in accordance with the manufacturer's instructions. As directed by the manufacturer, treated cells were tested for cell proliferation using the Click‐iT EdU‐488 Cell Proliferation Detection Kit (BL915A, Biosharp, Anhui, China) and DAPI (C1002, Beyotime, Shanghai, China). Images were obtained using an inverted microscope (BFM‐800E, Bimu, Shanghai, China).

### 2.4 Transwell Migration and Invasion Assays

#### 2.4.1 Migration

Following trypsinization and centrifugation, the treated cells were resuspended in basal media at a concentration of 2 × 10^3^ cells/mL per group. The lower chamber of a 24‐well Transwell plate was filled with 500 μL of full RPMI‐1640 media containing 10% FBS, and the upper chamber was filled with 200 μL of the cell suspension. The plate was then incubated for 24 h. Following incubation, phosphate buffered saline (PBS) was used to wash the chambers three times. The cells were exposed to with 4% paraformaldehyde (R20497, Yuanye, Shanghai, China) for 15 min, followed by three PBS washes. Following a 15‐min staining with 0.1% crystal violet (C0121, Beyotime, Shanghai, China), the cells were rinsed three times with PBS. Finally, the number of RA‐HFLS cells that migrated to the underside of the membrane in the chamber was quantified using an inverted microscope.

#### 2.4.2 Invasion

Combine Matrigel matrix gel with 1640 basal medium at a 1:8 ratio on ice. Fill the upper chamber of a 24‐well Transwell plate with 60 μL of the mixed matrix gel. The plate was incubated for 3 h. Following incubation, the previously described procedures were followed for the migration assay.

### 2.5 Pyroptosis

#### 2.5.1 Flow Cytometry and AO/EB Staining

The Annexin V‐FITC and propidium iodide (PI) Detection Kit (A5001‐02P‐L, Simu, Tianjin, China) was applied to stain the processed cells following the manufacturer's guidelines. A flow cytometer (BD FACS Calibur, BD Biosciences, USA) was subsequently employed to quantify pyroptosis.

Using the Acridine Orange/Ethidium Bromide (AO/EB) dual‐fluorescent staining kit (R20292, Genentech, Shanghai, China), treated cells were stained in accordance with the manufacturer's instructions. Pyroptosis was detected and captured using an inverted microscope.

#### 2.5.2 Immunofluorescence Staining (IF)

Cells were immobilized using 4% paraformaldehyde in 24‐well plates. After blocking with 3% BSA for 1 h, the cells were incubated overnight at 4°C with primary antibodies targeting NLRP3 (BA3677, Bosterbio, Wuhan, China) and IL‐1β (GB11113, Servicebio, Wuhan, China). Subsequently, the cells were incubated at room temperature for 50 min with secondary antibodies conjugated to the primary antibodies, without exposure to light. Lastly, DAPI (B0011, Baiqiandu Biotechnology, Wuhan, China) was used to stain the cell nuclei for 10 min. The cells were then sealed with an anti‐fluorescence quenching agent and stored in the dark at 4°C. Images were captured using an upright fluorescent microscope (Eclipse C1, Nikon, Japan).

### 2.6 Establishment of Animal Models and Treatment

#### 2.6.1 Animals and Grouping

Sixty female Wistar rats (weighing 180–200 g) were purchased from Yisi Animal Technology Co. Ltd. in Changchun City, Jilin Province, China. The exclusive use of female rats was based on the well‐established sex disparity in the epidemiology of rheumatoid arthritis (RA), with females being more commonly affected than males [29, 30], to enhance the clinical relevance and translational value of the findings. All animal experiments were conducted in accordance with the policies of the US Public Health Service on the use of laboratory animals. The rats were housed in a pathogen‐free environment with a 12‐h light/dark cycle at a temperature of 24°C ± 2°C. Every animal experiment was authorized by Changchun University of Chinese Medicine's Ethics Committee (Approval No. 2024841) and according to a set protocol. Six groups of 10 rats each were randomly selected from the 60 rats used in the experiment. The sample size of 10 rats per group was determined based on previously published studies using similar animal models of RA, in which this group size was sufficient to detect significant differences in key outcome measures such as paw volume, inflammatory cytokine levels, and histological scores [31, 32]. The groups included the control group (Con), CIA group, Tripterygium glycosides group (TG), high‐dose group (TY‐H), medium‐dose group (TY‐M), and low‐dose group (TY‐L).

#### 2.6.2 CIA Induction and Management

The establishment of the CIA rat model was established using a method previously employed in the laboratory [33]. Bovine type II collagen (20021, Chondrex, USA) was mixed with complete Freund's adjuvant (F5881, Sigma‐Aldrich, USA) in a 1:1 (v/v) ratio to prepare the emulsion. With the exception of the control group, the remaining rats were subcutaneously injected with 0.15 mL of the emulsion into the right hind limb for primary immunization (designated as Day 0) and received a booster injection of 0.1 mL of the same emulsion on Day 7. From Day 14 onwards, rats were gavaged once daily: the control group and CIA group received distilled water, while the treatment groups received the corresponding concentrations of the drug. The experiment continued until Day 42, after which the animals were euthanized and samples were collected in accordance with the ethical guidelines of the Animal Center of Changchun University of Chinese Medicine. Blood samples were collected from the abdominal aorta using a disposable vacuum blood collection tube and a blood collection tube containing citrate. The blood in the vacuum blood collection tube was allowed to stand at room temperature for 2 h, then centrifuged to collect the serum, which was stored for later use. After the experiment, the right hind joints of all rats were quickly removed, fixed in 4% paraformaldehyde, and stored for 24 h before further experiments.

### 2.7 Assessment of Severe Arthritis

The degree of arthritis was assessed by the arthritis score, hind paw toe volume, joint surface temperature, and acetone cold sensitivity stimulation. Measurements were taken every 7 days from Day 0 until the end of the experiment to assess the severity of arthritis. The arthritis index (AI) score was employed to evaluate the severity of arthritis in rats. The scoring criteria are outlined as follows [34]: 0 points indicate no swelling, 1 point indicates involvement of the interphalangeal joints, 2 points indicate mild swelling of the joints and toes, 3 points indicate obvious swelling of the toes and ankle joints, and 4 points indicate severe arthritis affecting the entire foot. The scores from the four categories were summed, with the maximum total score being 16, thus quantifying the overall severity of arthritis. An AI ≥ 4 is generally considered indicative of the successful establishment of the RA rat model. AI score was conducted in a blinded manner. Two independent researchers, unaware of the group allocation, scored the arthritis index based on the criteria outlined above, and the final score for each animal was calculated as the mean of the two individual scores. Experimental data were recorded by researchers who were informed of the group assignments.

The volume of the hind paw was measured using a paw volume meter (PV‐200, Tamei, Chengdu, China) on the right hind limb of rats in each group, by recording the displacement volume of each rat's right hind limb. An infrared thermography device (HT‐201, Hti, Gongguan, China) was used to measure the temperature of the joint surface of the right hind paw of female Wistar rats from the moment the first symptoms of inflammation were noticed. The rats were acclimatized to the device, which included a separate plexiglass enclosure. Acetone (500 μL) was gently sprayed onto the mid‐tarsal region of the hindfoot using a 1 mL syringe. The acetone rapidly spread across the proximal half of the tarsal surface. The number of times the rat licked and scratched the hindfoot within 5 min was observed. Each measurement was performed in triplicate, and the mean value was taken as an indicator of the cold‐sensitive pain response [35].

### 2.8 Organ Index, Erythrocyte Sedimentation Rate, and Histopathology

#### 2.8.1 Organ Index and Erythrocyte Sedimentation Rate

Following euthanasia at the end of the experiment, the thymus and spleen were promptly removed and weighed in order to calculate the thymus and spleen indices. The organ index for each organ was determined by dividing its weight (mg/g) by the animal's total body weight. Following blood collection with the citrate‐containing tubes mentioned above, the tubes were inverted three times and immediately placed in a dynamic erythrocyte sedimentation rate analyzer (ESR‐2040, THME, Chongqing, China) for a 1‐h measurement.

#### 2.8.2 Hematoxylin–Eosin Staining and Safranin O–Fast Green Staining

Following conventional protocols, histological changes were observed using hematoxylin and eosin (H&E, BA4025, BASO, Zhuhai, China) and safranin O–fast green (S&F, Solarbio, Beijing, China) staining. The procedure for H&E staining is outlined as follows: The paraffin sections were deparaffinized, hydrated, and then stained for 5 min with hematoxylin solution.

After washing with water, the sections were differentiated with hydrochloric ethanol differentiation solution, followed by counterstaining with eosin solution for 3 min. The tissue sections were then dehydrated in a gradient of alcohol, cleared with xylene, and finally mounted with neutral gum. The S&F staining procedure is outlined as follows: After deparaffinization and hydration of the sections as described above, they were stained with solid green solution for 5 min. After water washing and differentiation, the sections were counterstained with Orange G solution for 1 min. The sections were then dehydrated rapidly in four baths of absolute ethanol, and the remaining steps were the same as described above. Images were observed and captured using an optical microscope (BX51, Olympus, Japan). The outcome assessment was conducted in a blinded manner, in which two researchers, unaware of the group allocations, independently observed and described the pathological changes in each group.

#### 2.8.3 Immunohistochemistry

Following antigen retrieval, endogenous peroxidase inactivation, and blocking, tissue sections were incubated overnight at 4°C with primary antibodies against NLRP3 (1:100; GB114320, Servicebio, Wuhan, China) and IL‐1β (1:500; GB11113, Servicebio, Wuhan, China). Following washing, slices were incubated for 1 h at ambient temperature with a secondary antibody conjugated with horseradish peroxidase (HRP) (1:200; GB23303, Servicebio, Wuhan, China). Subsequently, sections were developed using diaminobenzidine (DAB), counterstained with hematoxylin, cleaned in xylene, dehydrated using a graded ethanol series, and mounted. Finally, immunostained sections were visualized and imaged using bright‐field microscopy.

### 2.9 LDH Release Assay

After processing the cells, the supernatant was obtained through centrifugation, and the LDH level in the supernatant was measured following the manufacturer's instructions (YX‐E10891, Youxuan, Shanghai, China). Additionally, the LDH level in rat serum was determined according to the manufacturer's instructions (A020‐2‐1, Jianchen, Nanjing, China).

### 2.10 ELISA

After completing the cell culture, the supernatant was collected by centrifugation. The amounts of IL‐1β (YX‐091203H), IL‐18 (YX‐E10092H), and TNF‐α (YX‐201407H) in the cell supernatant were determined using an enzyme‐linked immunosorbent assay (ELISA) kit (Shanghai Youxuan, China) in accordance with the manufacturer's instructions. Moreover, the levels of IL‐1β (YX‐091203R), IL‐18 (YX‐091218R), TNF‐α (YX‐201406R), anti‐CCP (YX‐030316R), and RF (YX‐180600R) in rat serum were determined using specific ELISA kits according to the manufacturer's guidelines. The microplate reader was operated according to the manual instructions, and the aforementioned kits were detected at their specified wavelengths.

### 2.11 Evaluation of Hepatic and Renal Function Parameters

The levels of alanine aminotransferas (ALT, C009‐2‐1), aspartate aminotransferase (AST, C010‐2‐1), creatinine (CRE, C011‐2‐1), and blood urea nitrogen (BUN, C013‐2‐1) in rat serum were measured using the reagent kits (Nanjing Jiancheng, China) according to the manufacturer's instructions, and detected with a microplate reader at the specified wavelengths.

### 2.12 RT‐qPCR

The expression of target molecule mRNA in the samples was assessed by reverse transcription quantitative PCR. RNA was extracted from rat synovial tissue and RA‐HFLS with an RNA extraction kit (DP419, Tiangen, Beijing, China). The extracted RNA was reverse transcribed into complementary DNA (cDNA) using a cDNA synthesis kit (AU341, Fullgold, Beijing, China), followed by amplification using a PCR amplification kit (FP217, Tiangen, Beijing, China). Briefly, 0.8 μL of cDNA, 0.6 μL of upstream and downstream primers (mixed upstream and downstream primers, 10 μM, see Table S1), and 10 μL of SYBR Green premix were mixed and adjusted with RNase‐free ddH₂O to a final volume of 20 μL. The reaction mixture was thoroughly mixed and then used for amplification.

The reaction program was set as follows: 95°C for 15 min for pre‐denaturation, followed by 40 cycles of amplification. Each cycle consisted of 95°C for 10 s for denaturation, 60°C for 30 s for annealing, and 72°C for 30 s for extension. The amplification reaction was performed using the CFX Connect Fluorescence Quantitative PCR Detection System (Bio‐Rad, USA).

### 2.13 Western Blot

Protein samples from each experimental group were collected, and an appropriate volume of the extracted protein samples was mixed with 5× loading buffer. The mixture was boiled for 10 min to denature the protein. Sodium dodecyl sulfate‐polyacrylamide gel electrophoresis (SDS‐PAGE) was subsequently performed to separate the proteins. After electrophoresis, the proteins were transferred to a polyvinylidene fluoride (PVDF) membrane and incubated at 4°C for 40 min. The PVDF membrane was retrieved, blocked with 5% skimmed milk, and stirred on a shaker at room temperature (20°C–25°C) for 2 h. After blocking, the membrane was washed and incubated overnight with the primary antibodies on a shaker at 4°C. The primary antibodies used were Apoptosis‐associated speck‐like protein containing a CARD (ASC) (1:500, WL02462, Wanlei, Shenyang, China), NLRP3 (1:2000, 30109‐1‐AP, Proteintech, Wuhan, China), Caspase‐1 (1:4000, 22915‐1‐AP, Proteintech, Wuhan, China), GSDMD (1:3000, 20770‐1‐AP, Proteintech, Wuhan, China), GSDMD‐NT (1:1000, PU224937, Abmart, Shanghai, China), IL‐1β (1:500, WL00891, Wanlei, Shenyang, China), and β‐actin (1:3000, AF0718, Affinity, USA). The membrane was then incubated with goat anti‐rabbit secondary antibody (1:5000, AS029, Abclonal, Wuhan, China) for 1 h. After washing, the membrane was treated with ECL reagents for chemiluminescent detection. The membrane was exposed and analyzed using ImageJ software.

### 2.14 Image Analysis and Quantification

All images were acquired under standardized exposure conditions and analyzed with ImageJ software (NIH, Bethesda, MD, USA). For cell migration and invasion assays, images of the Transwell membrane were captured with a light microscope. Following image threshold segmentation and binarization, the “Analyze Particles” function in ImageJ was used to quantify the migrated or invaded cells. For histopathological evaluation of rat ankle joints, images were captured from the joint cavity region at the tibia‐talus junction. To provide a comprehensive view of the histopathological changes, three images were captured from the joint cavity area at 100× magnification (Figure 6E). All image analyses were conducted by researchers blinded to the experimental groups.

### 2.15 UHPLC‐Q Exactive HFX

#### 2.15.1 Sample Preparation

1 mL of the high‐dose TY medicine solution was added to a centrifuge tube, followed by the addition of twice its volume of extraction solution (methanol/acetonitrile, 1:1, v/v). The solution was vortexed for 60 s, followed by ultrasonic extraction at low temperature for 30 min. The supernatant was recovered following centrifugation at 12 000 rpm for 10 min at 4°C. The proteins were then precipitated by incubating the supernatant at −20°C for 1 h. The supernatant was then collected for vacuum drying after a second centrifugation at 12 000 rpm for 10 min at 4°C. Subsequently, 100 μL of 50% acetonitrile solution was used for reconstitution. Following vortexing, the mixture was centrifuged for 10 min at 4°C at 12 000 rpm, and the supernatant was extracted for additional analysis. The conditions for liquid chromatography‐mass spectrometry are outlined in Tables S1 and S2.

#### 2.15.2 Data Preprocessing

The original information was analyzed with Progenesis QI software (Waters Corporation, Milford, USA) for alignment of peaks, adjustment of retention times, and identification of peak areas. The mass‐to‐charge ratio should be accurately matched (with a mass tolerance of fewer than 10 ppm), and the secondary spectra should be compared (with a mass tolerance of < 0.01 Da) to determine the metabolite composition.

### 2.16 Molecular Docking

The PubChem database (https://pubchem.ncbi.nlm.nih.gov/) was used to download 2D structures of small molecules. These were input into ChemOffice software to generate their 3D structures and save them as mol2 files. High‐resolution protein crystal structures were selected as receptors from the RCSB PDB database (http://www.rcsb.org/). PyMOL 2.6 software was used to remove water and phosphate groups from these structures before saving them as PDB files. AutoDock 1.5.6 was used for preprocessing of proteins and ligands, including hydrogenation and torsion force determination, while setting docking box coordinates. Molecular docking was performed using AutoDock Vina software. The optimal conformation was selected based on binding energy (lower binding energy indicates stronger binding activity). Finally, the docking results were visualized with the use of Discovery Studio 2019 and PyMOL 2.6, generating 2D and 3D interaction analysis diagrams.

### 2.17 Statistical Analysis

Data analysis wasconducted with GraphPad Prism 9.5 software. Data are expressed as the mean ± standard deviation. For comparisons between three or more groups, Tukey's test was applied following one‐way ANOVA. Statistical significance was set at a *p* value < 0.05.

## 3 Results

### 3.1 TY Attenuates the Regulatory Effects of TNF‐α on RA‐HFLS Cell Function

**FIGURE 1:**
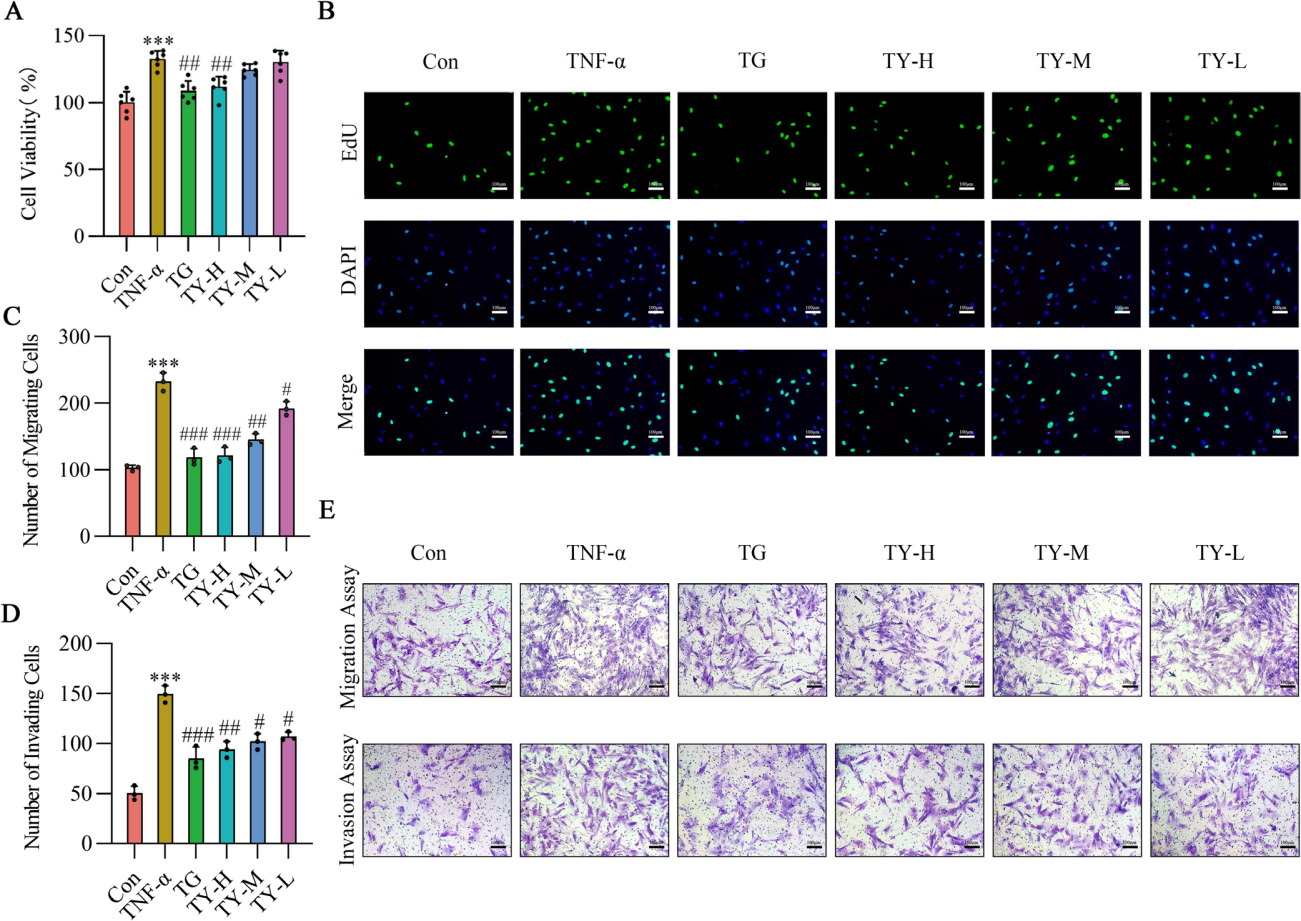
TY attenuates the regulatory effects of TNF‐α on RA‐HFLS cell function. (A) Cell viability of RA‐HFLS was evaluated using the CCK‐8 assay after stimulation with TNF‐α (24 h) with or without TY treatment (*n* = 6). (B) EdU incorporation assay showing the effect of TY on TNF‐α–induced RA‐HFLS proliferation; EdU‐positive nuclei (green) were counterstained with DAPI (blue). Images were captured at 100× magnification, scale bar = 100 μm (*n* = 3). (C, D) Quantification of Transwell migration and invasion (*n* = 3). (E) Representative images of migrated and invaded RA‐HFLS stained with crystal violet. Images were acquired at 100× magnification, scale bar = 100 μm. Data are presented as mean ± SD. ***p < 0.001 versus Con group; #*p* < 0.05, ##*p* < 0.01, ###*p* < 0.001 versus TNF‐α group (one‐way ANOVA). TNF‐α, tumor necrosis factor‐α; TY, TY formulation.

Initially, CCK‐8 and EdU tests were employed to assess the impact of serum formulations containing TY on the viability and proliferation of RA‐HFLS cells. These analyses demonstrated that serum containing TY effectively suppressed TNF‐α‐induced increases in cell viability and proliferation, as shown in Figure 1A,B. Additionally, transwell assays were performed to assess cell migration and invasion. Following treatment with serum containing TG and TY, a significant reduction in both migration and invasion was observed, indicating a marked decline in the migratory and invasive capabilities of RA‐HFLS cells, as shown in Figure 1C–E. Overall, these results indicate that TY inhibits the proliferation, migration, and invasion of RA‐HFLS cells.

### 3.2 TY Serum Inhibits NLRP3 Inflammasome‐Mediated Pyroptosis in RA‐HFLS Cells

**FIGURE 2:**
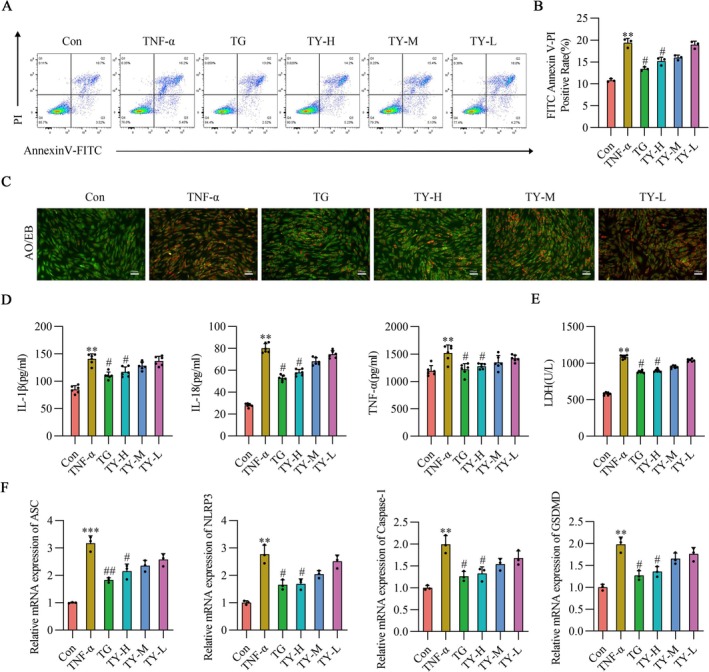
TY attenuates TNF‐α–induced pyroptosis and inflammatory responses in RA‐HFLS. (A) Representative flow cytometry plots showing pyroptosis. (B) Quantification of pyroptotic RA‐HFLS based on flow cytometry analysis (*n* = 3). (C) Representative images of AO/EB staining showing live (green) and pyroptotic (orange/red) RA‐HFLS after TNF‐α stimulation with or without TY treatment. Images were captured at 100× magnification, scale bar = 100 μm (*n* = 3). (D) ELISA analysis of IL‐1β, IL‐18, and TNF‐α secretion in culture supernatants (*n* = 6). (E) LDH release assay showing cell membrane damage (*n* = 6). (F) qPCR analysis of ASC, NLRP3, Caspase‐1, and GSDMD mRNA expression levels (*n* = 3). Data are presented as mean ± SD. **p < 0.01, *** *p* < 0.001 versus Con group; #*p* < 0.05, ##*p* < 0.01 versus TNF‐α group (one‐way ANOVA). TNF‐α, tumor necrosis factor‐α; TY, TY formulation.

The flow cytometry results are shown in Figure 2A,B, where TG and TY reduced the PI positivity rate in RA‐HFLS cells induced by TNF‐α. Since PI positivity is a marker of cell necrosis and membrane integrity disruption [36], these results suggest that TG and TY may reduce TNF‐α‐induced cell damage by maintaining cell membrane integrity. As shown in Figure 2C, the number of AO/EB positive cells in TNF‐α‐induced RA‐HFLS cells increased, but following intervention with TG and TY‐H serum, the number of AO/EB positive cells decreased. Notably, TG and TY serum decreased the levels of IL‐1β, IL‐18, TNF‐α, and LDH in the supernatant of TNF‐α‐induced RA‐HFLS cells, as shown in Figure 2D,E.

To further validate the above findings, we employed RT‐qPCR to detect the mRNA expression levels of ASC, NLRP3, Caspase‐1, and GSDMD in RA‐HFLS cells. The detailed primer sequences are provided in Table S3. RT‐qPCR results demonstrated that serum intervention with both TG and TY resulted in a significant decrease in the mRNA expression levels of these genes, as shown in Figure 2F.

**FIGURE 3:**
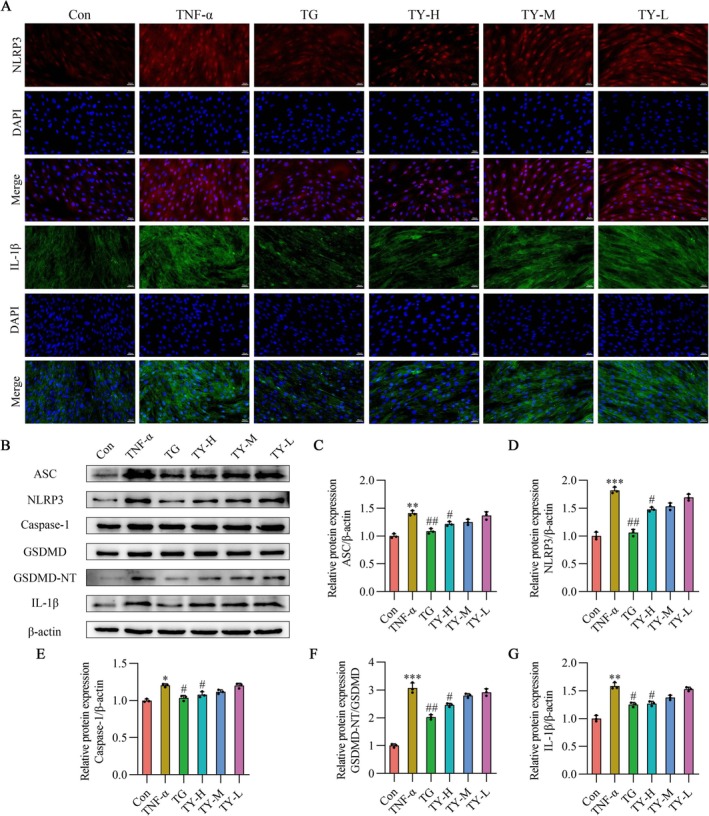
TY suppresses TNF‐α–induced activation of the NLRP3 inflammasome pathway in RA‐HFLS. (A) Representative immunofluorescence images showing the expression of NLRP3 (red) and IL‐1β (green) in RA‐HFLS. Nuclei were counterstained with DAPI (blue). Images were acquired at 100× magnification, scale bar = 100 μm (*n* = 3). (B) Representative Western blot bands of ASC, NLRP3, Caspase‐1, GSDMD, GSDMD‐NT, and IL‐1β. (C–G) Quantification of protein expression levels: ASC (C), NLRP3 (D), Caspase‐1 (E), GSDMD‐NT normalized to GSDMD (F), and IL‐1β (G) (*n* = 3). ASC, NLRP3, Caspase‐1, and IL‐1β were normalized to β‐Actin. Data are presented as mean ± SD. *p < 0.05, **p < 0.01, ***p < 0.001 versus Con group; #*p* < 0.05, ##*p* < 0.01 versus TNF‐α group (one‐way ANOVA). TNF‐α, tumor necrosis factor‐α; TY, TY formulation.

Subsequently, to investigate how serum containing TY treats pyroptosis in TNF‐α‐treated cells, we identified pyroptosis‐associated proteins through IF and Western blot analysis. IF revealed the presence of pyroptosis‐related proteins in RA‐HFLS cells. The results indicated that the expression levels of NLRP3 and IL‐1β in RA‐HFLS cells were elevated in comparison to the Con group. Figure 3A illustrates how TG or TY serum treatment decreased NLRP3 and IL‐1β expression in RA‐HFLS cells. Western blot analysis showed that the expression levels of ASC, NLRP3, Caspase‐1, IL‐1β, and GSDMD‐NT/GSDMD were significantly elevated in TNF‐α‐stimulated RA‐HFLS cells. The expression of these proteins was thereafter markedly downregulated upon treatment with serum containing TY, as Figure 3B–G illustrates.

### 3.3 TY Improves Arthritis Severity in CIA Rats

**FIGURE 4:**
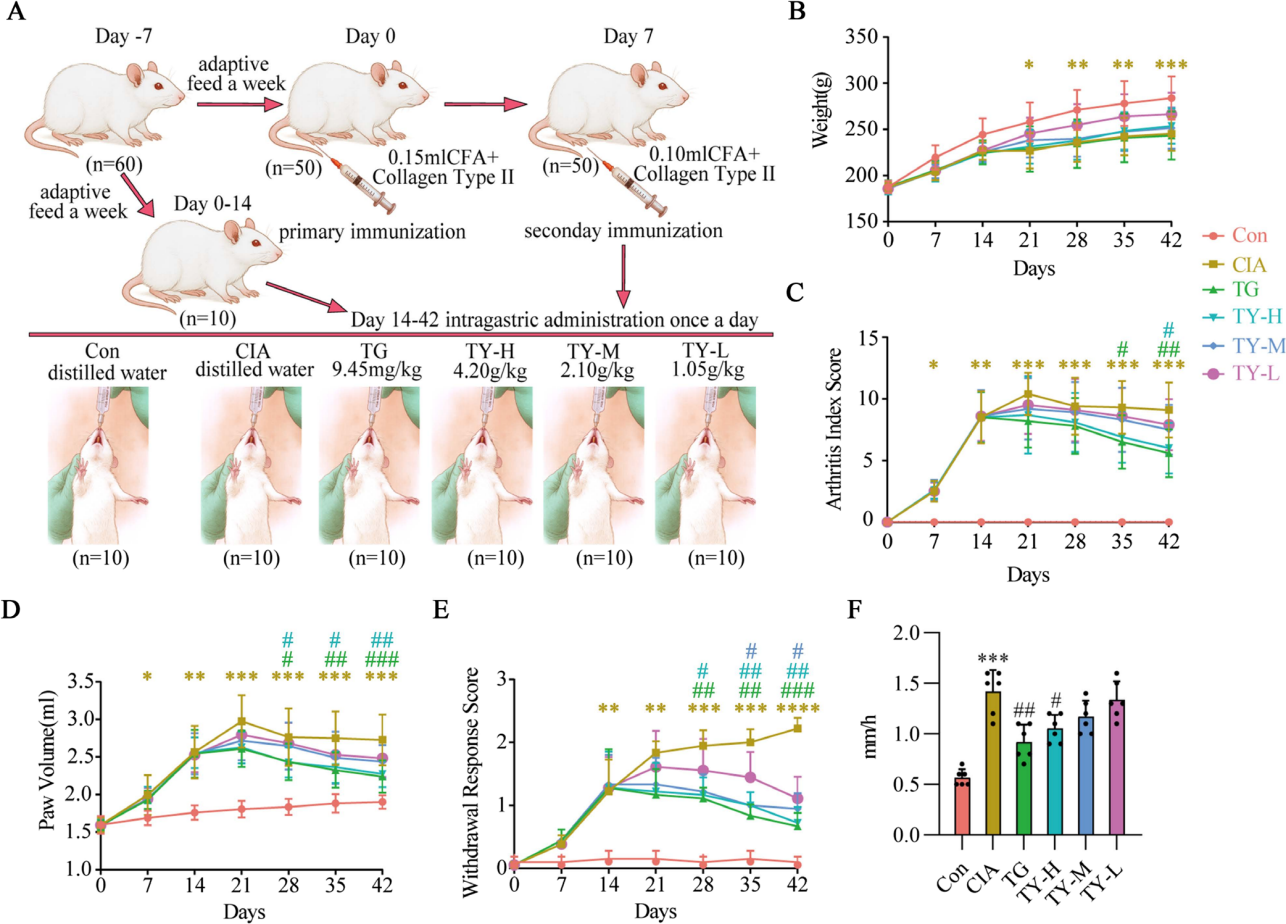
Effects of TY on clinical features in the CIA rat model. (A) Schematic diagram of the CIA modeling process. (B) Changes in body weight of rats during the experimental period (*n* = 10). (C) Arthritis index scores evaluated in different groups (*n* = 10). (D) Hind paw volume measurements showing joint swelling (*n* = 10). (E) Acetone cold allodynia test assessing cold sensitivity of hind paws (*n* = 6). (F) Erythrocyte sedimentation rate (ESR) of rats in each group (*n* = 6). Data are presented as mean ± SD. *p < 0.05, **p < 0.01, ***p < 0.001, ****p < 0.0001 versus Con group; #*p* < 0.05, ##*p* < 0.01, ###*p* < 0.001 versus CIA group (one‐way ANOVA). CIA, collagen‐induced arthritis; TY, TY formulation.

To assess the therapeutic efficacy of TY against RA, we successfully established a CIA model to simulate RA, following the specific procedures outlined in Figure 4A. Every rat gained weight in comparison to the starting point. Rats in the CIA group began to lose weight significantly on Day 21 when compared to the control group. As seen in Figure 4B, there were no statistically significant variations in body weight among the different drug‐treated groups as compared to the CIA group.

On Day 14, both the CIA group and the treatment groups exhibited joint redness, swelling, and even deformation (arthritis score ≥ 4), indicating successful establishment of the model [34]. Compared to the CIA group, all treatment groups showed a reduction in arthritis indices after administration, with the TG and TY‐H groups demonstrating more significant reductions on Days 35 and 42, as shown in Figure 4C.

Compared to the control group, rats in the CIA group and all treatment groups exhibited significant redness and swelling in the foot joints. Starting on Day 28, the TG and TY‐H groups showed significantly greater relief of joint redness, deformity, and swelling than the CIA group (*p* < 0.05). Symptoms also improved to varying degrees in the other treatment groups, as shown in Figure 4D. Additionally, CIA rats exhibited significantly greater hyperalgesia to acetone cold stimulation compared to the control group (*p* < 0.05). Starting from Day 28, the TG and TY‐H groups exhibited significantly fewer episodes of paw retraction/licking compared to the CIA group (*p* < 0.05). As observed in Figure 4E, symptoms in the other therapy groups also exhibited a tendency toward improvement. The CIA group displayed a significantly accelerated ESR rate compared to the control group. In contrast, both the TG and TY‐H groups demonstrated significantly reduced ESR rates relative to the CIA group, as depicted in Figure 4F.

**FIGURE 5:**
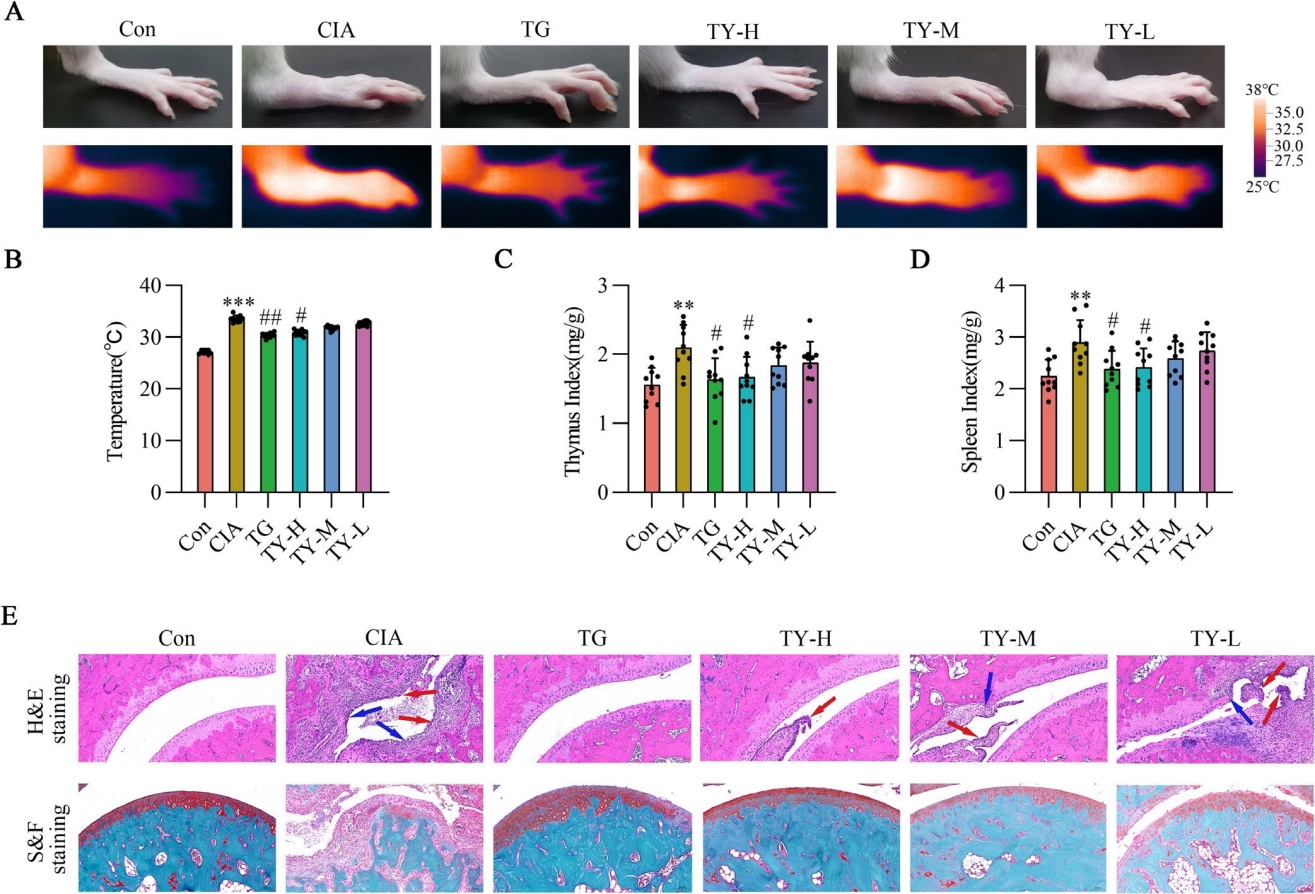
Effects of TY on thermoregulation, immune organ index, and histopathological changes in CIA rats. (A) Representative infrared thermographic images of hind paws. (B) Quantification of paw temperature in different groups (*n* = 10). (C, D) Thymus index (C) and spleen index (D) of rats (*n* = 10). (E) Representative histological images of ankle joints stained with hematoxylin–eosin (H&E) and Safranin O–fast green, showing synovial hyperplasia, inflammatory cell infiltration, and cartilage destruction. Red arrows indicate synovial hyperplasia, while blue arrows represent the destruction of the joint cavity or articular cartilage. Images were captured at 100× magnification, scale bar = 100 μm (*n* = 3). Data are expressed as mean ± SD. **p < 0.01, ***p < 0.001 versus Con group; #*p* < 0.05, ##*p* < 0.01 versus CIA group (one‐way ANOVA). CIA, collagen‐induced arthritis; TY, TY formulation.

Importantly, Figure 5A–D reveals that all TY therapies dramatically decreased thymus and spleen indices, increased heat‐induced hyperalgesia, and decreased disease severity. Concurrently, TY significantly reduced severe ankle swelling in CIA rats. Ankle slices from healthy rats display consistent joint gaps, undamaged joint surfaces, and a well‐organized layer of synovial cells covering the cartilage's outer surface. In contrast, synovial cells proliferate excessively in CIA rats, severely destroying the articular cartilage and joint cavity. Crucially, TY effectively ameliorated these pathological changes, which align with the pharmacological effects of TG, as shown in Figure 5E.

### 3.4 TY Inhibits NLRP3 Inflammasome‐Mediated Pyroptosis in CIA Rats

**FIGURE 6:**
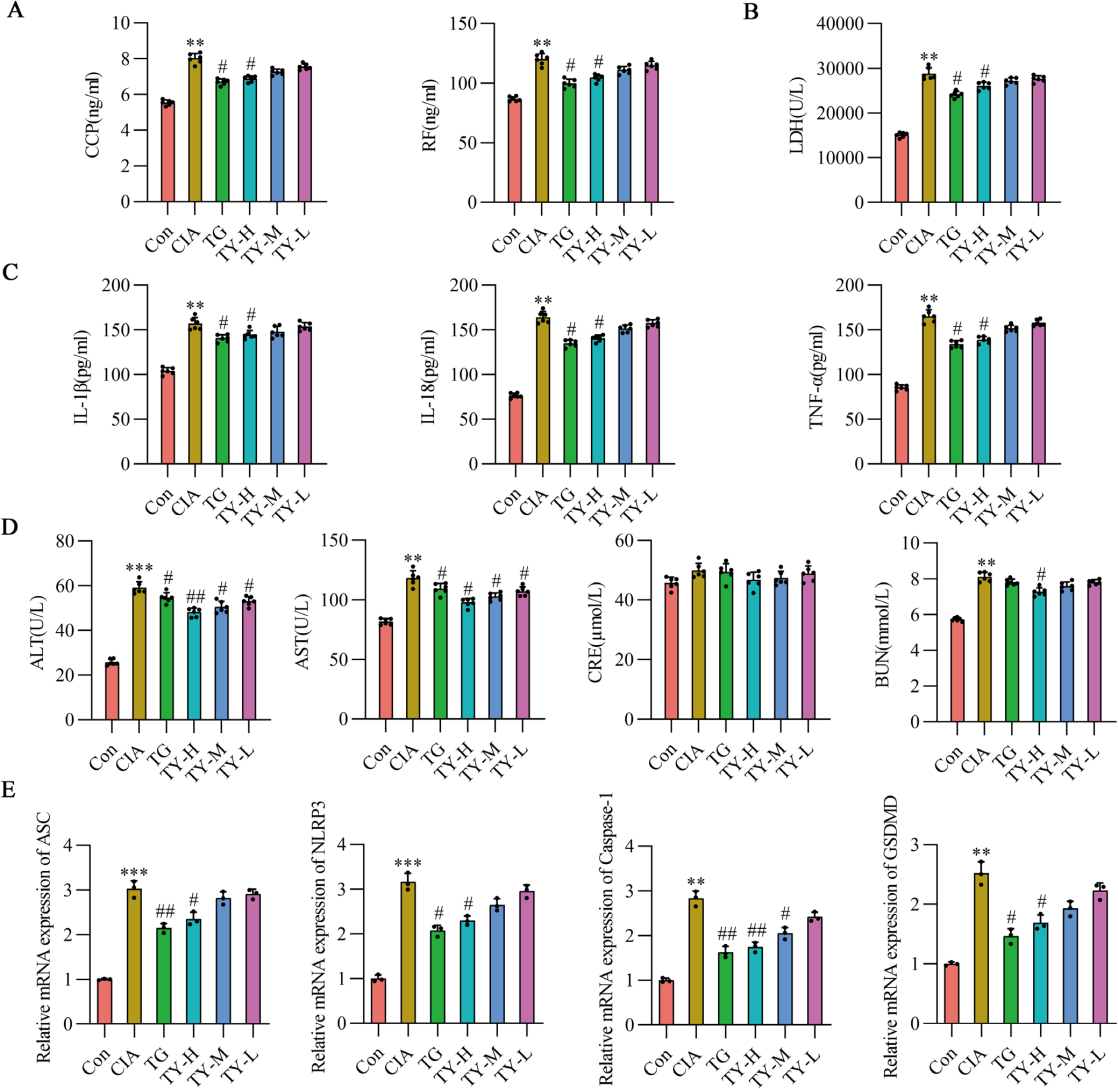
Effects of TY on serological markers, inflammatory cytokines, and inflammasome‐related gene expression in CIA rats. (A) Serum levels of anti‐cyclic citrullinated peptide antibody (anti‐CCP) and rheumatoid factor (RF) (*n* = 6). (B) Serum LDH levels (*n*=6). (C) ELISA analysis of serum IL‐1β, IL‐18, and TNF‐α levels (*n* = 6). (D) Serum levels of alanine aminotransferase (ALT), aspartate aminotransferase (AST), creatinine (CRE), and blood urea nitrogen (BUN) in each group (*n* = 6). (E) qPCR analysis of ASC, NLRP3, Caspase‐1, and GSDMD mRNA expression in joint tissues (*n* = 3). Data are expressed as mean ± SD. **p < 0.01, ***p < 0.001 versus Con group; #*p* < 0.05, ##*p* < 0.01 versus CIA group (one‐way ANOVA). CIA, collagen‐induced arthritis; TY, TY formulation.

Elevated anti‐CCP and RF titers were found in CIA rat serum when RA marker levels were assessed using ELISA assays. These results were reversed by TY intervention, as Figure 6A displays. LDH activity was used as an indicator of pyroptotic cell lysis and the release of intracellular soluble components. Consistently, treatment with TG and TY‐H resulted in a consistent reduction of increased LDH levels in CIA rat serum, as appeared in Figure 6B. Interestingly, the accumulation of GSDMD and the formation of membrane pores led to cell membrane rupture, triggering the release of IL‐1β and IL‐18—key hallmarks of pyroptosis. ELISA analysis of inflammatory factor release in rat serum revealed that IL‐1β, IL‐18, and TNF‐α levels were significantly higher in the CIA group compared to the control group. However, serum concentrations of these factors in the TG and TY‐H groups were significantly lower than in the CIA group, as depicted in Figure 6C. To evaluate the safety of TY, liver and kidney function indicators were assessed in rats. The results, shown in Figure 6D, demonstrated that all TY dosage groups significantly reduced the levels of ALT and AST in the serum of CIA rats, with the TY‐H group demonstrating particularly significant effects in lowering BUN levels in the serum of CIA rats. No statistically significant differences were observed among the dosage groups in terms of CRE levels. These results suggested that TY did not induce hepatotoxicity or nephrotoxicity within the experimental dose range.

Subsequently, our RT‐qPCR results showed that the synovial joints of CIA rats exhibited higher levels of ASC, NLRP3, Caspase‐1, and GSDMD mRNA expression. The detailed primer sequence information is provided in Table S4. Treatment with TY significantly reduced the mRNA expression levels of these proteins, demonstrating a pharmacological effect similar to that of TG, as indicated in Figure 6E.

**FIGURE 7:**
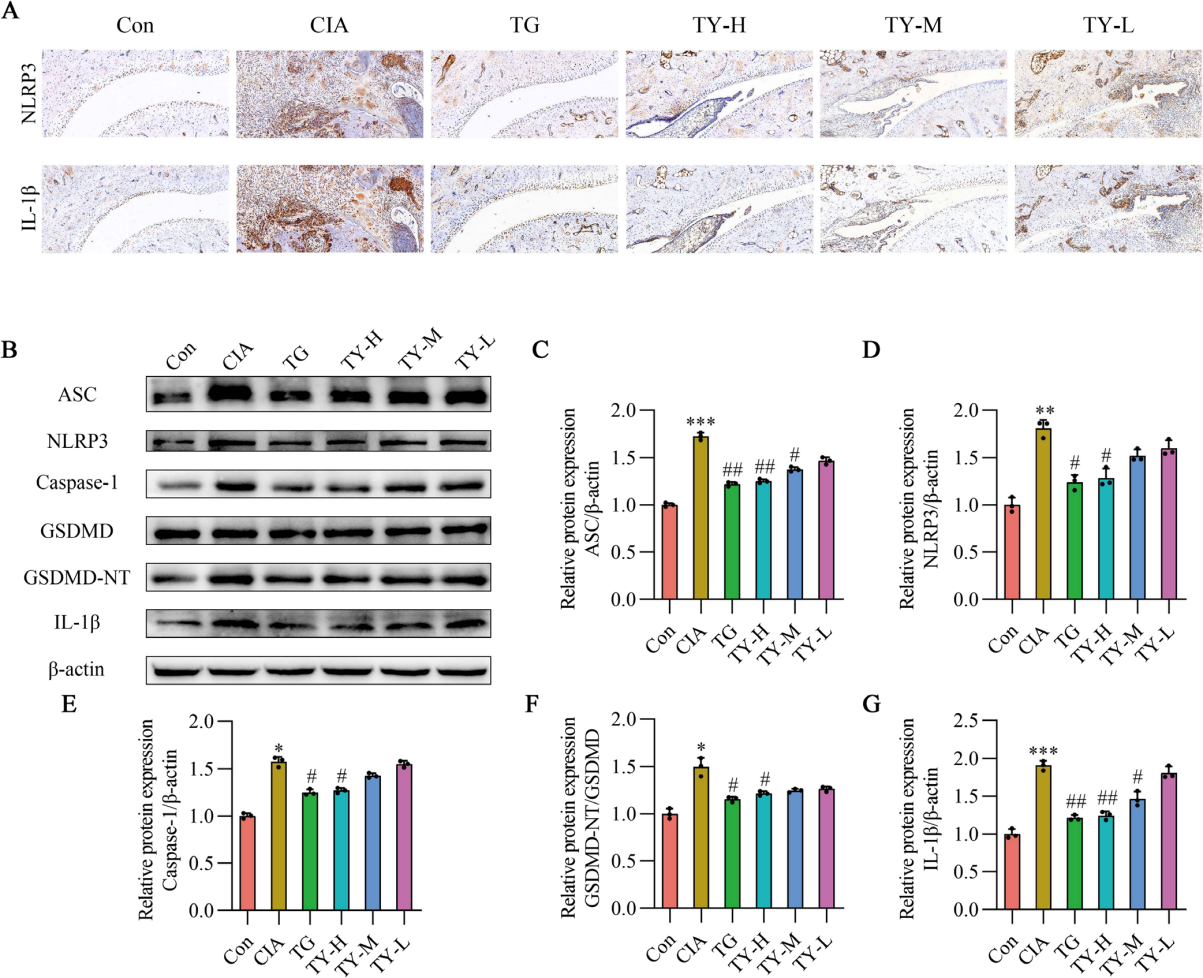
TY regulates NLRP3 inflammasome activation in CIA rat joints. (A) Representative immunohistochemistry images showing NLRP3 and IL‐1β expression in ankle joint sections. Images were captured at 100× magnification, scale bar = 100 μm (*n* = 3). (B) Representative Western blot bands of ASC, NLRP3, Caspase‐1, GSDMD, GSDMD‐NT, and IL‐1β. (C–G) Quantification of protein expression levels: ASC (C), NLRP3 (D), Caspase‐1 (E), GSDMD‐NT normalized to GSDMD (F), and IL‐1β (G) (*n* = 3). ASC, NLRP3, Caspase‐1, and IL‐1β were normalized to β‐actin. Data are expressed as mean ± SD. *p < 0.05, **p < 0.01, ***p < 0.001 versus Con group; #*p* < 0.05, ##*p* < 0.01 versus CIA group (one‐way ANOVA). CIA, collagen‐induced arthritis; TY, TY formulation.

To further investigate the inhibitory effect of TY on pyroptosis, immunohistochemical staining was performed to detect pyroptosis‐related proteins in synovial tissue. The NLRP3 and IL‐1β expression levels in CIA rats were considerably greater than those in the control group, as indicated by the results. Treatment with TG or TY effectively reduced the expression of NLRP3 and IL‐1β in synovial tissue, as shown in Figure 7A.

Furthermore, Western blot analysis revealed a significant increase in the expression of pyroptosis‐associated proteins in the synovial tissue of CIA rats. The expression of these proteins was significantly decreased by both TG and TY‐H treatments. As shown in Figure 7B,C, these results suggest that TY lowers the incidence of pyroptosis brought on by NLRP3.

### 3.5 Identification of Compounds in TY and Molecular Docking Analysis of Active Ingredients

**FIGURE 8:**
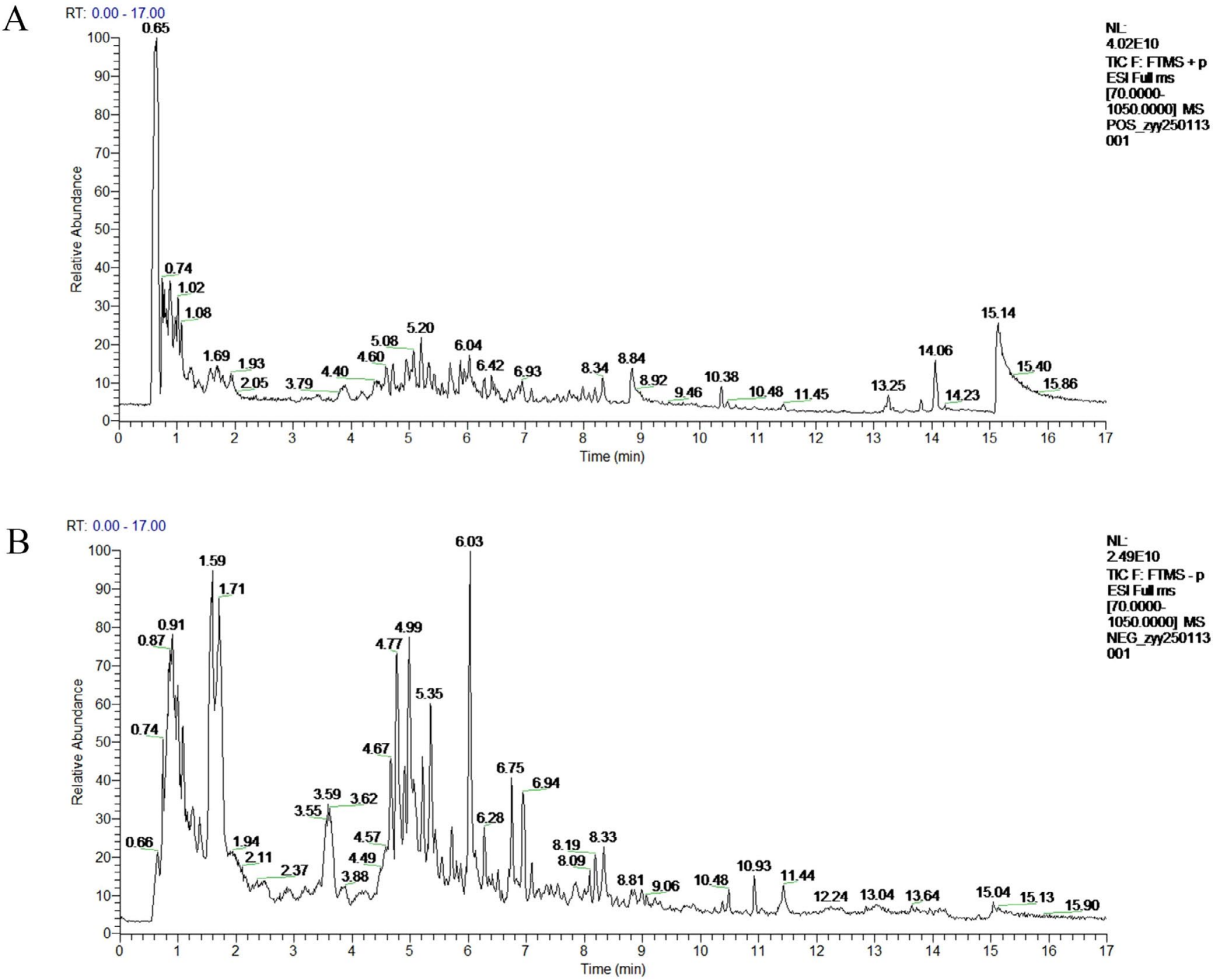
Total ion chromatograms (TIC) of TY formulation (TY) in positive and negative ionization modes. (A) TIC of TY acquired in the positive ion mode. (B) TIC of TY acquired in the negative ion mode.

The total ion chromatogram (TIC) of TY extracts was acquired in both positive and negative ion modes using UHPLC‐Q Exactive HFX, as depicted in Figure 8. A total of 95 compounds were identified, categorized into 16 groups, with phenylpropanoids and polyketides (42 compounds in total) constituting the dominant categories. This was followed by organic oxides (12 compounds), which were relatively abundant in the samples, while other categories contained fewer compounds. The identification of TY components was performed by comparing with reference compounds and matching empirical formulas and mass fragments to known compounds from the literature. The identification results of the TY components are provided in Table S5.

Previous studies have demonstrated that abnormal activation of the NLRP3/Caspase‐1/GSDMD pyroptosis pathway is closely associated with the pathogenesis of RA [37, 38, 39]. The aforementioned experiments confirmed that the TY compound can improve RA symptoms by regulating this pathway. However, the active components within TY compound targeting this pathway remain unclear. Therefore, based on UHPLC‐Q Exactive HFX mass spectrometry identification results, along with the Chinese Pharmacopeia's herbal material listings and literature reports on component associations with this pathway [40, 41, 42, 43, 44], five candidate compounds were identified: Apigenin, Isorhamnetin, Kaempferol, Quercetin, and Salidroside.

**FIGURE 9:**
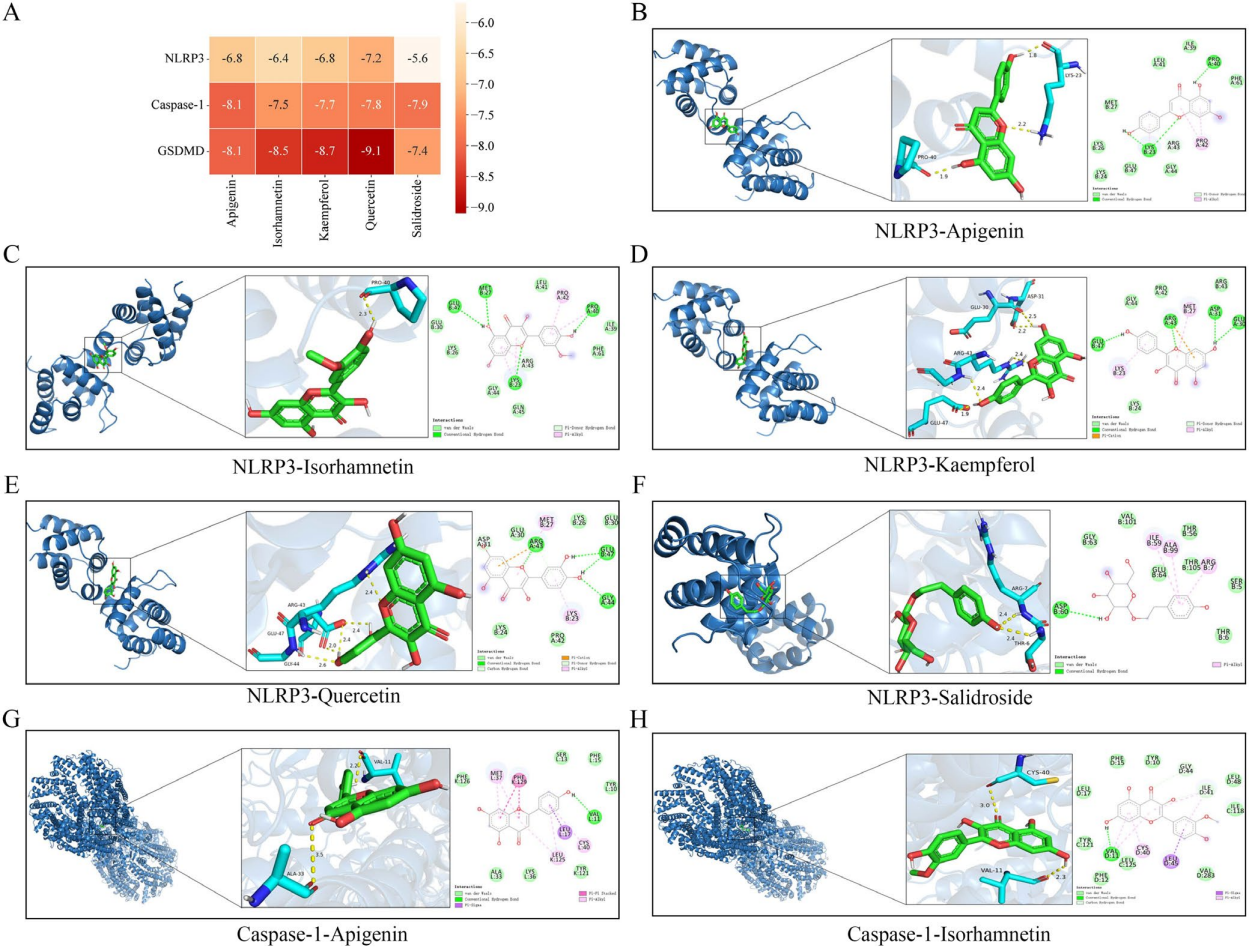
Molecular docking of core compounds with key target proteins. (A) Diagram showing the binding energy of molecular docking results. (B) NLRP3‐Apigenin, (C) NLRP3‐Isorhamnetin, (D) NLRP3‐Kaempferol, (E) NLRP3‐Quercetin, (F) NLRP3‐Salidroside, (G) Caspase‐1‐Apigenin, (H) Caspase‐1‐Isorhamnetin.

**FIGURE 10:**
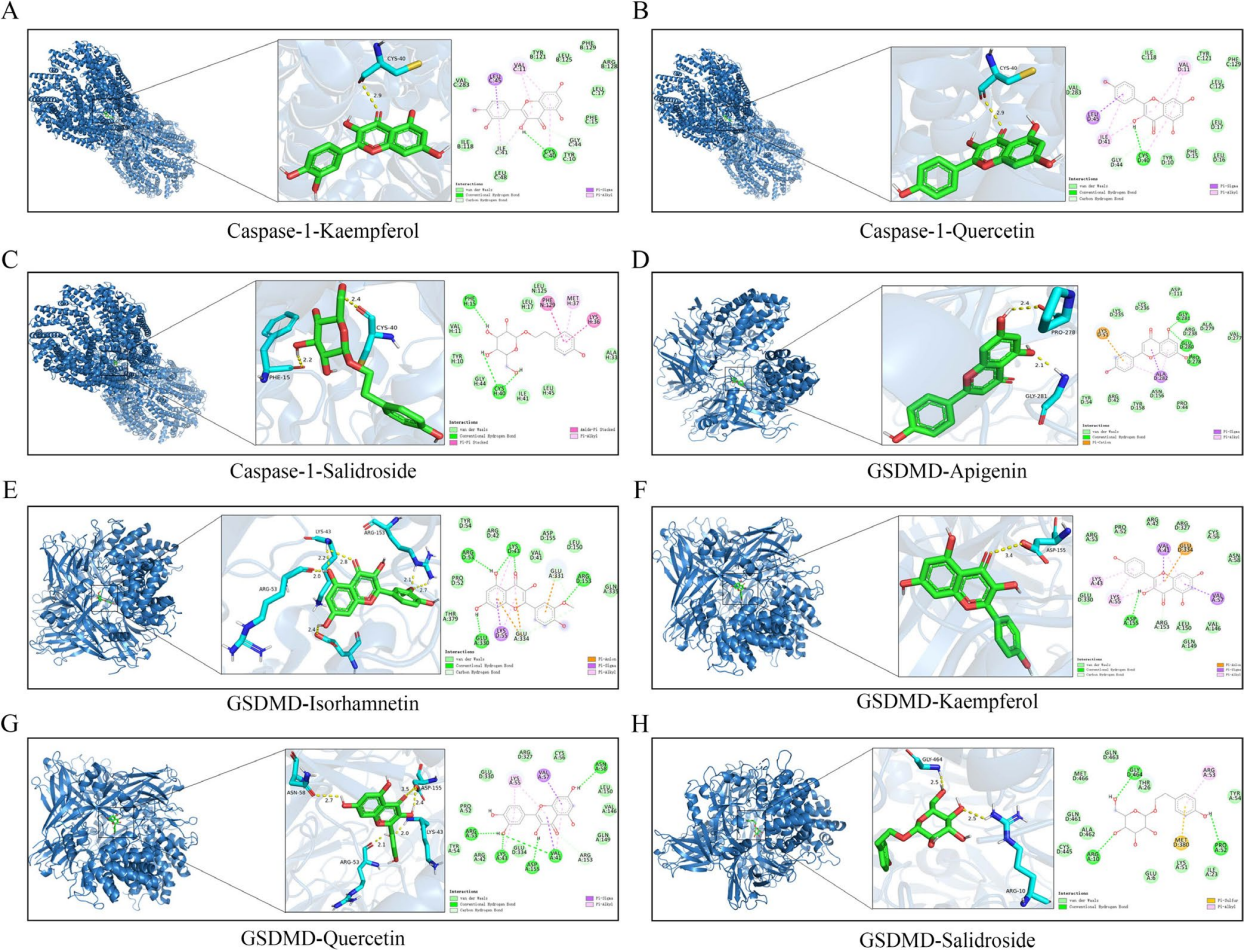
Molecular docking of core compounds with key target proteins. (A) Caspase‐1‐ Kaempferol, (B) Caspase‐1‐Quercetin, (C) Caspase‐1‐Salidroside, (D) GSDMD‐Apigenin, (E) GSDMD‐Isorhamnetin, (F) GSDMD‐Kaempferol, (G) GSDMD‐Quercetin, (H) GSDMD‐Salidroside.

To investigate the interactions between these five active components and key proteins in the NLRP3/Caspase‐1/GSDMD signaling pathway, molecular docking technology was used to dock each compound with the three targets: NLRP3, Caspase‐1, and GSDMD. The results are shown in Figure 9A. Generally, a binding energy below −5 kcal/mol indicates good binding affinity between the ligand and receptor. Docking results revealed that all five compounds exhibited binding energies below this threshold with the three targets, confirming their strong binding potential. Among them, quercetin showed the strongest binding affinity with GSDMD (−9.1 kcal/mol). Molecular docking models of the five compounds with their target proteins are shown in Figures 9B–H and 10A–H.

## 4 Discussion

In the pathological microenvironment of the synovium and synovial fluid in RA, cytokines such as TNF‐α, IL‐1, and IL‐6 released by synovial macrophages not only drive the inflammatory process but also stimulate the activation of FLS cells [45]. Activated RA‐FLS cells further secrete matrix metalloproteinases, various cytokines, and contribute to osteoclast activation and immune cell recruitment, collectively promoting the occurrence and development of cartilage degradation and bone erosion [46]. Notably, studies have demonstrated that pyroptosis regulates key genes, including AIM2, NLRP1, and PLCG1, activates NOD‐like receptor and NF‐κB signaling pathways, and induces a high‐inflammatory subtype (Cluster B) dominated by macrophages and Th17 cells, thereby significantly exacerbating the aforementioned pathological processes [47]. Therefore, by inhibiting pyroptosis, particularly in RA‐HFLS cells, and reducing the excessive activation of inflammatory cells, developing therapeutic approaches targeting these pathways may offer new breakthroughs in the treatment of RA.

This study utilized RA‐HFLS cells as the in vitro experimental model, and TNF‐α stimulation was applied to replicate the inflammatory microenvironment within the joint cavity. TNF‐α is a core factor driving the pathological progression of RA, which promotes the inflammatory storm and activates FLS cells by activating the NF‐κB pathway, ultimately leading to progressive joint destruction [48]. In RA, FLS cells undergo epigenetic regulation and phenotypic transformation, transforming into highly invasive cells [49]. These transformed cells not only have enhanced proliferation, migration, and invasion capabilities but also secrete large amounts of inflammatory factors, invading cartilage and bone tissue to form proliferative granulomas, thereby continuously exacerbating joint structural damage [49]. Experimental results showed that TY effectively reduced the viability, proliferation, migration, and invasion abilities of RA‐HFLS cells. In this process, we also observed the occurrence of pyroptosis, suggesting that pyroptosis could be a key contributor to the inflammatory processes in RA, a finding consistent with previous studies [50]. Flow cytometry and AO/EB staining results showed that under TNF‐α stimulation, the number of Annexin V/PI and AO/EB positive cells in RA‐HFLS cells increased, indicating pyroptosis occurrence. Pyroptosis is a type of programmed necrotic cell death mediated by the Gasdermin family, marked by cell membrane rupture and the release of inflammatory cytokines, along with the activation of the inflammatory response [51]. Under PAMP or DAMP stimulation, the NLRP3 inflammasome can be activated, leading to its assembly [52]. This complex consists of NLRP3, ASC, and Caspase‐1 precursors [53]. Upon recognition of the stimulus signal, NLRP3 binds to ASC, which promotes the oligomerization of ASC's CARD domain, which in turn recruits Caspase‐1 precursors [54]. The assembled inflammasome complex then mediates downstream signaling, and the recruited Caspase‐1 precursors are cleaved and activated, triggering the maturation of cytokines IL‐1β and IL‐18 and initiating pyroptosis mediated by GSDMD [55]. The cleaved GSDMD‐NT creates pores in the cell membrane, facilitating the release of mature IL‐1β and IL‐18, ultimately causing cell swelling and rupture [56]. This study found that TY can mitigate this pathological process. In vitro studies demonstrated that TY reduced the mRNA and protein expression of the NLRP3/Caspase‐1/GSDMD pathway in RA‐HFLS cells and decreased the levels of IL‐1β, IL‐18, TNF‐α, and LDH in the cell supernatant, thereby alleviating pyroptosis and the associated inflammatory response.

Subsequently, in order to more accurately simulate the complex autoimmune nature of RA, along with its typical clinical symptoms and pathological progression, the CIA rat model was successfully established. The rats in this model exhibited joint swelling, redness, and limited mobility, which closely resemble the clinical manifestations of human RA [57, 58]. Histopathological evaluation further demonstrated that the CIA rats exhibited chronic synovitis, significant infiltration of inflammatory cells, cartilage destruction, and bone erosion, which are typical pathological features of RA [59], suggesting that this model effectively replicates the core pathological processes of RA. Building on this, the results of our study demonstrate that after TY intervention, the arthritis score and ankle joint swelling in CIA rats were significantly reduced, while joint stiffness and mobility limitations were improved. Furthermore, the degree of pathological damage to the joint tissue was also notably alleviated. These results collectively suggest that TY effectively alleviates arthritis symptoms and joint structural damage in CIA rats, providing preliminary evidence of its potential to improve RA‐related inflammation and tissue destruction. Previous studies have shown that the expression of NLRP3, Caspase‐1, and GSDMD is upregulated in the synovial tissue of CIA rats, with increased levels of IL‐1β and IL‐18, indicating that pyroptosis plays an important role in the occurrence and progression of synovial damage in CIA joints [60]. Pyroptosis primarily exacerbates the deterioration of the local inflammatory microenvironment through the release of inflammatory factors [61]. Simultaneously, it disrupts cellular homeostasis and impairs normal physiological processes, such as cellular metabolism and signal transduction, further intensifying the inflammatory response in RA [62]. Recent studies have found that, compared to healthy controls, the expression of NLRP3, Caspase‐1, and GSDMD‐NT is significantly upregulated in the synovial tissue of RA patients. Moreover, the levels of IL‐1β, IL‐18, and LDH in the synovial fluid are also markedly increased, and these indicators are positively correlated with disease activity and inflammation levels, indicating that pyroptosis plays a crucial role in the pathogenesis of RA [63]. Notably, small molecule drugs targeting the inhibition of the NLRP3 inflammasome (such as ZYIL1 and OLT‐1177) have demonstrated therapeutic potential in clinical studies, further validating the therapeutic value of regulating the pyroptosis pathway [64]. These findings suggest that pyroptosis plays a significant role in the chronic inflammatory response in RA. This study further observed that TY could downregulate the expression of these pyroptosis‐related molecules and reduce the release of IL‐1β and IL‐18, suggesting that TY effectively alleviates the pyroptosis‐related pathological changes in CIA rats. This result is consistent with previous studies [32, 65]. Additionally, the effect of TY on liver and kidney function was also assessed. The results showed that all TY dosage groups significantly reduced the levels of ALT, AST, and BUN in the serum of CIA rats to varying degrees. This suggests that while TY exerts therapeutic effects against RA, it does not cause damage to the liver or kidneys. Along with literature reports on the liver and kidney protective effects of *Rhodiola crenulata* and *Euonymus alatus* , the results of this study further confirm that TY, as a compound formulation, possesses good safety, providing experimental evidence supporting its clinical application [66, 67, 68, 69]. This study further supports that TY, as a compound preparation, demonstrates a favorable safety profile, providing experimental evidence supporting its future clinical application.

In vitro and in vivo results demonstrate that TY can effectively alleviate inflammation and synovial damage in CIA through modulation of the NLRP3/Caspase‐1/GSDMD pathway, thereby improving the overall pathology of RA. This demonstrates its potential clinical value and provides experimental evidence supporting its use as an intervention for RA. However, the specific components in TY responsible for these effects remain unclear. Therefore, this study employed UHPLC‐Q Exactive HFX technology to preliminarily identify the chemical constituents in the TY formulation, identifying 95 compounds in total and clarifying its primary chemical composition. Based on these findings, and in conjunction with literature reports and entries in the Chinese Pharmacopeia, five active components potentially associated with the NLRP3/Caspase‐1/GSDMD pyroptosis pathway were selected for molecular docking analysis. Molecular docking results showed that the binding energies of all five active components with NLRP3, Caspase‐1, and GSDMD were below −5 kcal/mol, indicating a stable binding potential with the target proteins. Among them, quercetin showed the lowest binding energy (−9.1 kcal/mol) and the highest affinity with GSDMD, suggesting that it may be one of the key active molecules in the TY compound that regulates this pathway. Quercetin has been shown to inhibit the abnormal proliferation and invasion of RA‐FLS and reduce the release of inflammatory cytokines, thus effectively alleviating joint inflammation and bone destruction in RA [70]. Similarly, apigenin exerts an immunosuppressive effect by inhibiting the maturation and migration of dendritic cells, thus alleviating joint inflammation and bone destruction in CIA mice and reducing serum levels of pro‐inflammatory factors such as TNF‐α, IL‐1β, and IL‐6 [71]. Apigenin has also been shown to inhibit synovial hyperplasia, angiogenesis, and osteoclast activation, thus exerting a protective effect on CIA mice [72]. Additionally, it has been shown that isorhamnetin effectively inhibits the proliferation, migration, and invasion of RA‐FLS cells and reduces the levels of pro‐inflammatory factors such as TNF‐α, IL‐6, and IL‐1β [73]. Interestingly, kaempferol inhibits pyroptosis in synovial tissue cells by regulating the NLRP3/Caspase‐1/GSDMD axis, reducing the levels of pro‐inflammatory factors such as TNF‐α, IL‐1β, IL‐6, and IL‐18, and upregulating the anti‐inflammatory factor IL‐10, thus alleviating joint inflammation and bone destruction [74]. Moreover, salidroside promotes osteoblast differentiation, improves bone microstructure, enhances antioxidant capacity, and inhibits the expression of TNF‐α and IL‐6, thereby exerting anti‐inflammatory effects [75]. In summary, quercetin, apigenin, kaempferol, isorhamnetin, and salidroside may be the core active components of the TY compound that regulate the NLRP3/Caspase‐1/GSDMD pyroptosis pathway. This finding provides a molecular‐level mechanistic explanation for the intervention of the TY compound in RA and provides a theoretical basis for the screening and structural optimization of active components based on the TY compound.

Although this study systematically elucidates the mechanism by which TY alleviates pyroptosis in RA synovial cells through the inhibition of the NLRP3/Caspase‐1/GSDMD pathway, certain limitations remain. Firstly, one key limitation of this study is the lack of a single‐herb control group for *Rhodiola rosea* and *Euonymus alatus* . Therefore, although the compound formulation demonstrated significant therapeutic efficacy, the available data are not sufficient to determine whether the observed activity results from a synergistic effect, an additive effect, or the dominant action of a single herb. This limitation prevents definitive conclusions regarding the traditional formulation theory of the compound and highlights directions for future research. Future studies should employ methods such as the Chou‐Talalay combined index or isoradiation analysis to perform a component‐by‐component analysis of the compound, thus rigorously verifying the synergistic effects proposed by traditional medical theory.

Secondly, although in vitro experiments using RA‐HFLS cells confirmed the upregulation of NLRP3 and IL‐1β under experimental conditions, in vivo studies were assessed only by immunohistochemical staining, without performing immunofluorescence colocalization analysis. While immunohistochemistry can reveal the distribution and expression levels of NLRP3 and IL‐1β in tissues, it cannot accurately pinpoint the specific cellular origins of these proteins. Therefore, the exact cellular origin of inflammasome activity in joint tissues remains unclear. Although RA‐HFLS cells may be involved, it remains unclear whether other immune cells, such as macrophages and lymphocytes, play a role in the tissue microenvironment. Future studies should employ techniques such as multiplex immunofluorescence staining, flow cytometry, or single‐cell RNA sequencing to further clarify the cellular origin of inflammasome activation in this model.

Thirdly, although this study comprehensively identified the soluble components in the TY decoction through chemical analysis and screened five active compounds for molecular docking, the absorption and biological activity of these components in vivo remain unclear. Therefore, future studies should integrate targeted quantitative methods (such as LC–MS/MS) to detect the key biomarkers identified in this study and incorporate serum pharmacology studies to characterize the absorbed components and their metabolites in the TY compound. These efforts are essential for bridging the critical gap between the chemical profile of the decoction and the actual pharmacologically active substances in vivo.

## 5 Conclusion

In conclusion, this study shows that TY reduces pyroptosis in RA‐HFLS cells and ameliorates joint inflammation in CIA rats by mitigating the NLRP3/Caspase‐1/GSDMD pathway.

## Author Contributions

Yinghang Wang and Zhi Pan conceived and designed the research; Yueya Zhu and Xinying Cai performed the research and acquired the data; Qiuhan Zheng and Chunqiu Fang analyzed and interpreted the data; Yueya Zhu and Yinghang Wang were involved in drafting and revising the manuscript. All authors review this manuscript and agree to submit it for publication.

## Funding

This work was supported by the Health Commission of Jilin Province (2025ZY‐ZA009) and the Jilin Provincial Scientific and Technological Development Program (JJKH20250645KJ).

## Ethics Statement

This study was approved by the Ethics Committee of Changchun University of Chinese Medicine (Approval Nos. 2024841 and 2024842).

## Conflicts of Interest

The authors declare no conflicts of interest.

## Supporting information


**Table S1:** Liquid chromatography parameters.
**Table S2:** Mass spectrometry conditions.
**Table S3:** Primer sequences used for cell experiments.
**Table S4:** Primer sequences used for rat experiments.
**Table S5:** Identification of the chemical constituents of *Rhodiola crenulata* and *Euonymus alatus* extract by UHPLC‐Q Exactive HFX.
**Figure S1:** Original western blot images for in vitro (cellular) experiments.
**Figure S2:** Original western blot images for in vivo (animal) experiments.

## Data Availability

The data that support the findings of this study are available on request from the corresponding authors.
