# Supplementary material for: The “Tianyu” Formulation Alleviates Rheumatoid Arthritis by Modulating the NLRP3/Caspase‐1/GSDMD‐Mediated Pyroptosis Pathway
Source: FASEB J. 2026 Apr 25;40:e71824. doi: 10.1096/fj.202504611R (PMC13109814; doi:10.1096/fj.202504611R)

Supplementary Table 1: Liquid Chromatography Parameters

| Parameter Category | Details |
| --- | --- |
| Column | Waters HSS T3 (100×2.1 mm, 1.8 μm, 50%m) |
| Mobile Phase | Phase A: ultrapure water with 0.1% formic acid; Phase B: acetonitrile with 0.1% formic acid |
| Flow Rate | 0.3 mL/min |
| Column Temperature | 40℃ |
| Injection Volume | 2 μL (50%L) |
| Elution Gradient | 0 min: A/B = 100:0 (v/v)  1 min: A/B = 100:0 (v/v)  12 min: A/B = 5:95 (v/v)  13 min: A/B = 5:95 (v/v)  13.1 min: A/B = 100:0 (v/v)  17 min: A/B = 100:0 (v/v) |

Supplementary Table 2: Mass Spectrometry Conditions

| Parameter Category | Details |
| --- | --- |
| Ion Source | Electrospray ionization (ESI) |
| Sheath Gas | 40 arb |
| Auxiliary Gas | 10 arb |
| Spray Voltage | +3000 V / –2800 V |
| Ion Source Temperature | 350℃ |
| Ion Transfer Tube Temperature | 320℃ |

Supplementary Table 3. Primer sequences used for cell experiments

| Gene | Forward Primer (5′→3′) | Reverse Primer (5′→3′) |
| --- | --- | --- |
| ASC | AGTTCAAGCTGAAGCTGCTGTCG | CGCCGTAGGTCTCCAGGTAGAAG |
| NLRP3 | GATCTTCGCTGCGATCAACAG | CGTGCATTATCTGAACCCCAC |
| Caspase-1 | GTGCAGGACAACCCAGCTAT | GCAGGCCTGGATGATGATCA |
| GSDMD | GTGTGTCAACCTGTCTATCAAGG | CATGGCATCGTAGAAGTGGAAG |
| β-actin | GTCGTACCACTGGCATTGTG | TCTCAGCTGTGGTGGTGAAG |

Supplementary Table 4. Primer sequences used for rat experiments

| Gene | Forward Primer (5′→3′) | Reverse Primer (5′→3′) |
| --- | --- | --- |
| ASC | GGCACAGCCAGAACAGAACATT | TGTTTTGGTTGGGGGTCTCT |
| NLRP3 | GGTTGGTGAATTCCGGCCTTACT | CTTACAGTCGGGGTGCAGAAGTC |
| Caspase-1 | GTCTGTGGGCAGGAAGTGAA | TCCCTGTTTCTTCAGTGTGGG |
| GSDMD | CTGGTGTTGTCCTCCGGAAT | CTGCGTTTCACTCAGCATGG |
| β-actin | CCCTGGAGAAGAGCTACGAG | GGAAGGAAGGCTGGAAGAGT |

Supplementary Table 5. Identification of the chemical constituents of Rhodiola crenulata and Euonymus alatus extract by UHPLC-Q Exactive HFX.

| No. | Identification | TR (min) | Formula | Calculated mass | Observed mass | Mass Error (ppm) | Adducts | MS Fragmentation | Categories | Herbs |
| --- | --- | --- | --- | --- | --- | --- | --- | --- | --- | --- |
| 1 | L-Arginine | 0.7419833 | C6H14N4O2 | 175.11895 | 175.11968 | 4.207287 | M+H | 158.0930,130.0982,116.0714,70.0660,60.0565 | Amino acid | Rhodiola crenulata |
| 2 | L-GLUCOSE | 0.8136333 | C6H12O6 | 179.05611 | 179.0566 | 2.7220051 | M-H | 161.0457,113.0246,101.0246,71.0140,59.0139 | Organic oxygen compounds | Rhodiola crenulata |
| 3 | N-Acetylglutamic acid | 0.86955 | C7H11NO5 | 188.05645 | 188.05703 | 3.0668273 | M-H | 144.0667,142.0510,114.0561,98.0249 | Organic acids and derivatives | Rhodiola crenulata |
| 4 | Neolinustatin | 0.8710833 | C17H29NO11 | 465.20769 | 465.2099 | 4.7600648 | M+ACN+H | 465.2094,303.1558,250.1081,235.1101 | Organic oxygen compounds | Rhodiola crenulata |
| 5 | Citric acid | 0.91935 | C6H8O7 | 191.01973 | 191.0202 | 2.4834107 | M-H | 191.0226,173.0110,129.0187,111.0091 | Aromatic acid | Rhodiola crenulata |
| 6 | L-Tyrosine | 0.9319 | C9H11NO3 | 164.07067 | 164.07128 | 3.7060798 | M+H-H2O | 164.0712,147.0442,136.0761,124.0240 | Organic acids and derivatives | Rhodiola crenulata |
| 7 | Adenine | 0.96515 | C5H5N5 | 136.06177 | 136.06231 | 3.9848272 | M+H-2H2O, M+H | 136.0623,119.0353,94.0414 | Organoheterocyclic compounds | Rhodiola crenulata |
| 8 | Guanine | 0.97335 | C5H5N5O | 152.05668 | 152.05728 | 3.9279214 | M+H, 2M+H, M+K | 152.0572,135.0307 | Organoheterocyclic compounds | Rhodiola crenulata |
| 9 | NICOTINIC ACID | 1.0097833 | C6H5NO2 | 122.02475 | 122.02504 | 2.3716975 | M-H, M+FA-H | 122.0226,121.0295,78.0350 | Organoheterocyclic compounds | Rhodiola crenulata |
| 10 | L-Leucine | 1.0734333 | C6H13NO2 | 132.1019 | 132.10244 | 4.0627883 | M+H | 132.1020,114.0921,97.0290,86.0972 | Amino acid | Rhodiola crenulata |
| 11 | Guanosine | 1.3315333 | C10H13N5O5 | 284.09894 | 284.10023 | 4.5426269 | M+H | 152.0572,135.0307,85.0292 | Nucleosides, nucleotides, and analogues | Rhodiola crenulata |
| 12 | Gallic acid | 1.5789333 | C7H6O5 | 169.01425 | 169.01451 | 1.5697588 | M-H, 2M-H | 169.0152,125.0246,97.0296,79.0187 | Benzenoids | Rhodiola crenulata、Euonymus alatus |
| 13 | Adenosine | 1.6968333 | C10H13N5O4 | 268.10403 | 268.10484 | 3.0380194 | M+H | 268.1082,136.0622,120.0814 | Nucleosides, nucleotides, and analogues | Rhodiola crenulata |
| 14 | Quinic acid | 1.8003833 | C7H12O6 | 173.04558 | 173.04591 | 1.9076858 | M-H2O-H | 129.0556,115.0389,111.0453 | Organic oxygen compounds | Rhodiola crenulata |
| 15 | 2-Hydroxy-3-methoxybenzoic acid glucose ester | 2.484 | C14H18O9 | 329.08781 | 329.0892 | 4.2207232 | M-H | 167.0352,152.0115,123.0453,108.0233 | Organic oxygen compounds | Rhodiola crenulata |
| 16 | 5,7-Dihydroxychromone | 2.6086833 | C9H6O4 | 223.02463 | 223.02552 | 3.9662231 | M+FA-H | 179.0353,151.0400,137.0245,135.0453, | Organoheterocyclic compounds | Rhodiola crenulata |
| 17 | 1-O-Caffeoylglucose | 2.9475833 | C15H18O9 | 341.08781 | 341.08918 | 4.0123601 | M-H | 179.0343,135.0453 | Phenylpropanoids and polyketides | Rhodiola crenulata |
| 18 | 1-(4-Hydroxybenzoyl)glucose | 3.17515 | C13H16O8 | 299.07724 | 299.07825 | 3.3638949 | M-H | 299.0781,179.0351,137.0246,93.0348 | Organic oxygen compounds | Rhodiola crenulata |
| 19 | Epigallocatechin | 3.4755333 | C15H14O7 | 305.06668 | 305.06787 | 3.8898374 | M-H | 219.0671,179.0354,167.0351,125.0245 | Phenylpropanoids and polyketides | Rhodiola crenulata |
| 20 | Protocatechuic acid | 3.6005667 | C7H6O4 | 153.01933 | 153.01959 | 1.6718612 | M-H, M+Cl, 2M-H | 153.0194,109.0295,108.0217 | Benzenoids | Rhodiola crenulata |
| 21 | L-Tryptophan | 3.9197167 | C11H12N2O2 | 203.0826 | 203.08321 | 3.0069453 | M-H | 186.0560,159.0926,142.0663,116.0506 | Amino acid | Rhodiola crenulata |
| 22 | Rosavin | 4.4529167 | C20H28O10 | 409.1505 | 409.1525 | 4.8830452 | M-H2O-H | 165.0559,149.0244,71.0139,59.0139 | Lipids and lipid-like molecules | Rhodiola crenulata |
| 23 | Methyl gallate | 4.4847 | C8H8O5 | 183.0299 | 183.03037 | 2.5652512 | M-H | 168.0066,124.0167 | Benzenoids | Rhodiola crenulata |
| 24 | p-Coumaric acid | 4.5743 | C9H8O3 | 147.04411 | 147.04462 | 3.4489727 | M+H-H2O | 147.0045,119.0496,91.0549 | Phenylpropanoids and polyketides | Rhodiola crenulata、Euonymus alatus |
| 25 | 1,6-Digalloylglucose | 4.61515 | C20H20O14 | 483.07803 | 483.08041 | 4.9085457 | M-H | 271.0459, 211.0250,169.0143,125.0245 | Organic oxygen compounds | Rhodiola crenulata |
| 26 | p-Vinylphenyl O-beta-D-glucopyranoside | 4.6648667 | C14H18O6 | 247.09661 | 247.09749 | 3.5580732 | M+H-2H2O | 229.0867,201.0920,183.0809,157.0655 | Organic oxygen compounds | Rhodiola crenulata |
| 27 | Salidroside | 4.6739333 | C14H20O7 | 318.15467 | 318.15573 | 3.3508044 | M+H-2H2O, M+H-H2O, M+NH4, M+H, M+K | 179.0606,119.0494,89.0241 | Organic oxygen compounds | Rhodiola crenulata |
| 28 | 5-Hydroxymethylfurfural | 4.77235 | C6H6O3 | 127.03897 | 127.0396 | 4.9758105 | M+H | 127.0394,109.0289,81.0344, | Organic oxygen compounds | Euonymus alatus |
| 29 | Ferulic acid | 4.7883 | C10H10O4 | 193.05063 | 193.0511 | 2.3905446 | M-H | 178.0273,134.0374 | Phenylpropanoids and polyketides | Rhodiola crenulata、Euonymus alatus |
| 30 | Procyanidin B3 | 4.81555 | C30H26O12 | 561.13922 | 561.14187 | 4.7259083 | M+H-H2O, M+NH4 | 543.1283,409.0938,393.0983,259.0609 | Phenylpropanoids and polyketides | Euonymus alatus |
| 31 | (+)-Pinoresinol | 4.8334833 | C20H22O6 | 341.13841 | 341.13952 | 3.2660636 | M+H-H2O, M+H, M+H-2H2O | 341.1409,323.1295,311.1291,137.0602 | Lignans, neolignans and related compounds | Euonymus alatus |
| 32 | Protocatechualdehyde | 4.8888333 | C7H6O3 | 275.05612 | 275.05707 | 3.4773734 | 2M-H | 275.0188,137.0244 | Organic oxygen compounds | Euonymus alatus |
| 33 | Brevifolincarboxylic acid | 5.0540833 | C13H8O8 | 310.05568 | 310.05676 | 3.4961274 | M+NH4 | 293.0327,275.0573,219.0295,175.0401 | Phenylpropanoids and polyketides | Rhodiola crenulata |
| 34 | fraxin | 5.2068333 | C16H18O10 | 369.08272 | 369.08429 | 4.2425758 | M-H | 207.0300,192.0066,163.0039 | Phenylpropanoids and polyketides | Rhodiola crenulata |
| 35 | Isodemethylwedelolactone | 5.2772 | C15H8O7 | 301.03428 | 301.03539 | 3.6946986 | M+H | 273.0397,217.0508,147.0447,121.0286 | Phenylpropanoids and polyketides | Rhodiola crenulata |
| 36 | Vanillic acid glucoside | 5.3446333 | C14H18O9 | 311.0773 | 311.07834 | 3.3206854 | M-H2O-H | 267.0894,165.0561,137.0609 | Glycoside | Rhodiola crenulata |
| 37 | Aesculetin | 5.36455 | C9H6O4 | 179.03388 | 179.03468 | 4.4783549 | M+H | 151.0396,133.0291,123.0447 | Phenylpropanoids and polyketides | Rhodiola crenulata |
| 38 | Caffeic acid | 5.36455 | C9H8O4 | 181.04953 | 181.05025 | 3.9858703 | M+H-H2O, M+H | 135.0446,117.0341 | Phenylpropanoids and polyketides | Rhodiola crenulata、Euonymus alatus |
| 39 | Vanillic acid | 5.3654833 | C8H8O4 | 167.03498 | 167.03537 | 2.3063128 | M-H, 2M-H | 152.0114,123.0462,108.0216,91.0187 | Benzenoids | Euonymus alatus |
| 40 | quercitrin | 5.4224667 | C21H20O11 | 449.10783 | 449.10963 | 3.991162 | M+H | 449.0969,303.0505 | Phenylpropanoids and polyketides | Rhodiola crenulata、Euonymus alatus |
| 41 | (+)-Catechin hydrate | 5.4413167 | C15H14O6 | 291.0863 | 291.08718 | 3.0188373 | M+H-H2O, M+H | 291.0875,207.0659,139.0394,123.0446 | Flavonoid | Rhodiola crenulata、Euonymus alatus |
| 42 | Syringic acid | 5.4839167 | C9H10O5 | 197.04555 | 197.04617 | 3.1599346 | M-H | 182.0223,166.9989,153.0560,138.0323 | Benzenoids | Euonymus alatus |
| 43 | Astragalin | 5.5348833 | C21H20O11 | 449.10783 | 449.10981 | 4.3939659 | M+H | 449.1057,287.0557,153.0186 | Phenylpropanoids and polyketides | Rhodiola crenulata |
| 44 | 6'-O-Galloylsalidroside | 5.5653 | C21H24O11 | 451.12459 | 451.12592 | 2.9511479 | M-H, 2M-H, M+Cl | 169.0144,124.0166 | Benzenoids | Rhodiola crenulata |
| 45 | Procyanidin B2 3''-O-gallate | 5.5945667 | C37H30O16 | 731.16066 | 731.16397 | 4.5284647 | M+H | 409.0939,287.0559,127.0395 | Phenylpropanoids and polyketides | Rhodiola crenulata |
| 46 | Dihydromyricetin | 5.6487833 | C15H12O8 | 321.06049 | 321.06172 | 3.8244742 | M+H | 321.0607,195.0296,153.0188, | Phenylpropanoids and polyketides | Euonymus alatus |
| 47 | Kaempferol 3-rungioside | 5.6815333 | C27H30O15 | 595.16574 | 595.16831 | 4.3096716 | M+H | 287.0558 | Phenylpropanoids and polyketides | Rhodiola crenulata |
| 48 | Spiraeoside | 5.6913 | C21H20O12 | 465.10275 | 465.10472 | 4.2294012 | M+H | 303.0507,257.0456,229.0507,165.0193 | Phenylpropanoids and polyketides | Rhodiola crenulata |
| 49 | Benzoic acid | 5.7257 | C7H6O2 | 121.02951 | 121.02977 | 2.1864884 | M-H | 93.0349,92.0269,78.0375, | Aromatic acid | Euonymus alatus |
| 50 | Kaempferol | 5.7504333 | C15H10O6 | 287.05501 | 287.05609 | 3.7702768 | M+H | 287.0552,165.0187,153.0187,121.0186 | Phenylpropanoids and polyketides | Rhodiola crenulata、Euonymus alatus |
| 51 | 7,8,3',4'-tetrahydroxyflavanone | 5.9336 | C15H12O6 | 577.13405 | 577.1364 | 4.0820957 | 2M+H | 425.0868,287.0558,245.52 | Phenylpropanoids and polyketides | Euonymus alatus |
| 52 | Sinapic acid | 5.99775 | C11H12O5 | 225.07575 | 225.07666 | 4.0590255 | M+H | 207.0662,179.0706,151.0395,123.1174 | Phenolic acid | Rhodiola crenulata |
| 53 | Phyllanthurinolactone | 6.0462 | C14H18O8 | 279.08644 | 279.08739 | 3.4391542 | M+H-2H2O | 163.0398,153.0554 | Organic oxygen compounds | Rhodiola crenulata |
| 54 | Ellagic acid | 6.0536333 | C14H6O8 | 300.99899 | 300.99983 | 2.7699708 | M-H | 283.9966,245.0085,229.0132,2010181,185.0237,173.0238 | Phenylpropanoids and polyketides | Rhodiola crenulata、Euonymus alatus |
| 55 | Ethyl gallate | 6.0670333 | C9H10O5 | 197.04555 | 197.04607 | 2.6536469 | M-H | 169.0142,124.0166 | Benzenoids | Rhodiola crenulata |
| 56 | epicatechin gallate | 6.1057333 | C22H18O10 | 443.09727 | 443.09894 | 3.7755514 | M+H | 291.0870,153.0185,139.0394,123.0445 | Phenylpropanoids and polyketides | Rhodiola crenulata |
| 57 | (-)-Epicatechin gallate | 6.1146333 | C22H18O10 | 441.08272 | 441.08384 | 2.5332944 | M-H, M+Cl, 2M-H | 289.0723,245.0834,169.01432,125.0245 | Phenylpropanoids and polyketides | Rhodiola crenulata |
| 58 | Isovanillin | 6.1367833 | C8H8O3 | 153.05462 | 153.05517 | 3.6343437 | M+H, M+ACN+H | 135.1173,125.0595,107.0862,93.0342 | Benzenoids | Rhodiola crenulata |
| 59 | Cycloolivil | 6.1707833 | C20H24O7 | 341.13847 | 341.13963 | 3.4073422 | M+H-2H2O | 323.1291,311.1289,291.1026,263.1109 | Lignans, neolignans and related compounds | Rhodiola crenulata |
| 60 | Rosin | 6.20265 | C15H20O6 | 297.13326 | 297.13441 | 3.8770595 | M+H-H2O, M+H | 297.1181,261.1121,183.0294,153.0185 | Lipids and lipid-like molecules | Rhodiola crenulata |
| 61 | Hibifolin | 6.36375 | C21H18O14 | 495.07693 | 495.07908 | 4.3423262 | M+H | 495.0788,319.0457,301,0346 | Phenylpropanoids and polyketides | Rhodiola crenulata |
| 62 | Purpurogallin | 6.36915 | C11H8O5 | 265.03519 | 265.03628 | 4.1226986 | M+FA-H | 221.0466,203.0354,193.0509,153.0117 | Hydrocarbon derivatives | Rhodiola crenulata |
| 63 | Taxifolin | 6.3748333 | C15H12O7 | 305.06558 | 305.0667 | 3.6984384 | M+H | 305.0648,287.0564,259.0609,231.0661,153.0188 | Flavonoid | Rhodiola crenulata、Euonymus alatus |
| 64 | Azelaic acid | 6.4299833 | C9H16O4 | 187.09759 | 187.09808 | 2.6341337 | M-H | 187.1002,169.0867,125.0972,97.0662 | Lipids and lipid-like molecules | Rhodiola crenulata |
| 65 | Myricitrin | 6.4818167 | C21H20O12 | 465.10275 | 465.10481 | 4.4351702 | M+H | 319.0458,273.0392,153.0186 | Phenylpropanoids and polyketides | Rhodiola crenulata |
| 66 | Diosmetin-7-O-Beta-D-glucopyranoside | 6.5582333 | C22H22O11 | 463.12348 | 463.1255 | 4.3551497 | M+H | 301.0715,286.0479,258.0534 | Phenylpropanoids and polyketides | Rhodiola crenulata |
| 67 | Orientin 2''-O-p-trans-coumarate | 6.7171833 | C30H26O13 | 577.13412 | 577.13636 | 3.8845218 | M+H-H2O | 425.0887,245.0454 | Phenylpropanoids and polyketides | Rhodiola crenulata |
| 68 | Rutin | 6.7171833 | C27H30O16 | 611.16066 | 611.16291 | 3.6861222 | M+H | 303.0504,274.0475,153.0185 | Phenylpropanoids and polyketides | Rhodiola crenulata、Euonymus alatus |
| 69 | Myricetin | 6.8072833 | C15H10O8 | 319.04484 | 319.04582 | 3.0740877 | M+H | 273.0396,245.0454,165.0190,153.0188 | Phenylpropanoids and polyketides | Euonymus alatus |
| 70 | Isoquercitrin | 6.8303667 | C21H20O12 | 465.10275 | 465.10434 | 3.415367 | M+H | 465.1042,303.0508 | Phenylpropanoids and polyketides | Rhodiola crenulata |
| 71 | Secoisolariciresinol | 6.86955 | C20H26O6 | 361.16567 | 361.16721 | 4.2672915 | M-H | 361.1656,346.1444,179.0715,165.0559 | Lignans, neolignans and related compounds | Euonymus alatus |
| 72 | Beta-D-glucopyranosiduronic acid | 6.9396167 | C21H20O11 | 447.09329 | 447.09535 | 4.6012721 | M-H, 2M-H, M+Cl | 301.0356 | Aldonic acid | Rhodiola crenulata |
| 73 | Quercetin 7-rhamnoside | 6.9404167 | C21H20O11 | 449.10784 | 449.1086 | 1.697576 | M+H | 303.0502,257.0449,169.0137 | Phenylpropanoids and polyketides | Rhodiola crenulata |
| 74 | (-)-Lariciresinol | 7.0097167 | C20H24O6 | 361.16456 | 361.16592 | 3.7723636 | M+H-H2O, M+H | 285.1130,189.0918,181.00871,167.0702,161.0605 | Lignans, neolignans and related compounds | Euonymus alatus |
| 75 | Loliolide | 7.2212833 | C11H16O3 | 241.10798 | 241.10887 | 3.6744556 | M+FA-H | 197.1185,1791068,153.1281 | Organoheterocyclic compounds | Euonymus alatus |
| 76 | Taxifolin 7-rhamnoside | 7.29555 | C21H22O11 | 431.09845 | 431.10029 | 4.2671675 | M-H2O-H | 285.0409,257.0462,151.0038 | Phenylpropanoids and polyketides | Rhodiola crenulata |
| 77 | afzelin | 7.3047667 | C21H20O10 | 433.11292 | 433.11409 | 2.7071025 | M+H | 287.0556,241.0487 | Phenylpropanoids and polyketides | Rhodiola crenulata |
| 78 | Eriodictyol | 7.4634333 | C15H12O6 | 289.07066 | 289.07172 | 3.6595602 | M+H | 289.0717,163.0396,153.0188,135.0447 | Phenylpropanoids and polyketides | Rhodiola crenulata |
| 79 | Quercetin | 7.5339 | C15H10O7 | 303.04993 | 303.05085 | 3.0422962 | M+H | 303.0504,285.0373,229.0504 | Phenylpropanoids and polyketides | Rhodiola crenulata、Euonymus alatus |
| 80 | Acacetin | 7.56865 | C16H12O5 | 283.0612 | 283.06219 | 3.4835951 | M-H | 283.0623,268.0382,240.0426,239.0353,,211.0404 | Phenylpropanoids and polyketides | Rhodiola crenulata |
| 81 | (-)-Epigallocatechin-3-(3''-O-methyl) gallate | 7.7481667 | C23H20O11 | 455.09734 | 455.09908 | 3.832521 | M+H-H2O | 181.0500,179.0346,163.0399,139.0393,123.0448 | Phenylpropanoids and polyketides | Rhodiola crenulata |
| 82 | 7-Methoxycoumarin | 7.7635833 | C10H8O3 | 221.04534 | 221.04635 | 4.5886054 | M+FA-H | 177.0552,161.0458,133.0661 | Phenylpropanoids and polyketides | Rhodiola crenulata |
| 83 | Ophiopogonanone E | 7.8728333 | C19H20O7 | 361.12818 | 361.12957 | 3.8533191 | M+H | 329.1037,151.0394 | Phenylpropanoids and polyketides | Rhodiola crenulata |
| 84 | Apigenin | 8.0658167 | C15H10O5 | 271.0601 | 271.06121 | 4.0968377 | M+H | 271.0603,270.0904,229.0481,153.0188,119.0498 | Phenylpropanoids and polyketides | Rhodiola crenulata |
| 85 | Isorhamnetin | 8.1926 | C16H12O7 | 315.05103 | 315.05235 | 4.1952388 | M-H | 300.0289,272.0336,165.9909 | Phenylpropanoids and polyketides | Rhodiola crenulata |
| 86 | Herbacetin | 8.4302333 | C15H10O7 | 605.09258 | 605.09546 | 4.7707062 | 2M+H | 303.0507,273.0401,153.0183 | Phenylpropanoids and polyketides | Rhodiola crenulata |
| 87 | Palmitic acid | 8.8359833 | C16H32O2 | 274.274 | 274.27482 | 2.9786995 | M+NH4 | 274.2747,256.2645, | Lipids and lipid-like molecules | Rhodiola crenulata |
| 88 | Demethylwedelolactone | 8.9374667 | C15H8O7 | 299.01973 | 299.02081 | 3.619686 | M-H | 271.0255,215.0353,199.0405,171.0455 | Phenylpropanoids and polyketides | Rhodiola crenulata |
| 89 | Diosmetin | 9.71425 | C16H12O6 | 301.07066 | 301.07185 | 3.9395838 | M+H | 301.0724,286.0418,258.0537 | Phenylpropanoids and polyketides | Rhodiola crenulata |
| 90 | Alpha-Linolenic acid | 10.09405 | C18H30O2 | 296.25833 | 296.25959 | 4.257776 | M+NH4 | 279.2332,109.1019,81.0707,67.0551 | Lipids and lipid-like molecules | Rhodiola crenulata |
| 91 | PHYTOSPHINGOSINE | 10.21125 | C18H39NO3 | 300.28977 | 300.29084 | 3.5822994 | M+H-H2O | 300.2906,286.0461,282.2803 | Organic nitrogen compounds | Rhodiola crenulata |
| 92 | Malic acid | 10.848433 | C4H6O5 | 133.01425 | 133.01456 | 2.3150209 | M-H | 132.8678,115.0037,89.0256,71.0156 | Organic acids and derivatives | Rhodiola crenulata |
| 93 | 2-O-Methyluridine | 11.596067 | C10H14N2O6 | 239.06742 | 239.06832 | 3.778999 | M-H2O-H | 239.0679,223.0300,207.0417,179.0538 | Nucleosides, nucleotides, and analogues | Rhodiola crenulata |
| 94 | D-altrofurano-heptulose-3 | 14.0551 | C7H14O7 | 245.04321 | 245.0442 | 4.0399285 | M+Cl | 209.0661,149.0458,119.0350,101.0245 | Organic oxygen compounds | Rhodiola crenulata |
| 95 | Oleamide | 14.070583 | C18H35NO | 563.55186 | 563.55318 | 2.3314612 | 2M+H, M+ACN+H | 282.2803,247.2432 | Lipids and lipid-like molecules | Rhodiola crenulata |

Supplementary Figures:

Fig.S1 Original Western Blot Images for In Vitro (Cellular) Experiments


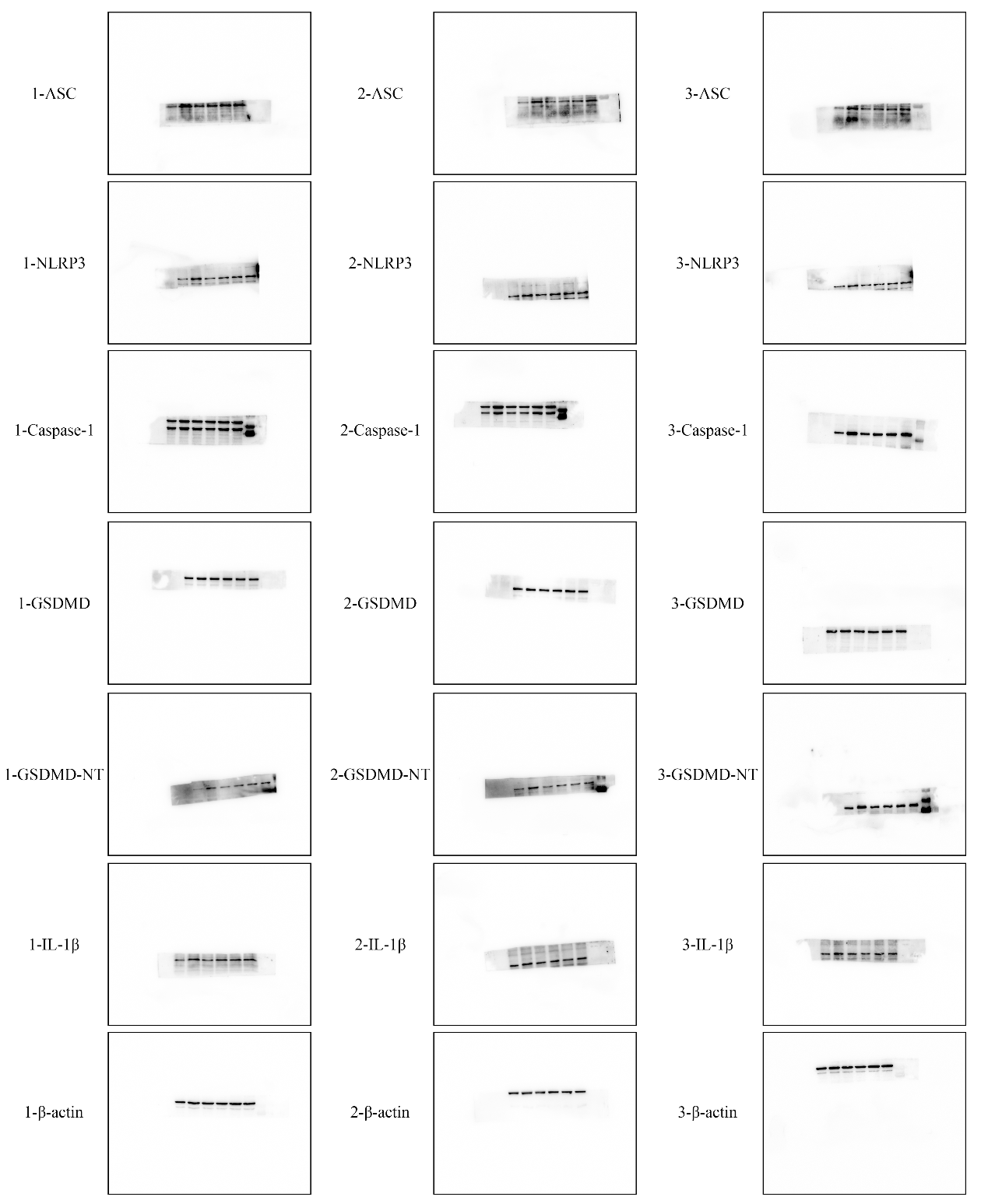


Fig.S2 Original Western Blot Images for In Vivo (Animal) Experiments


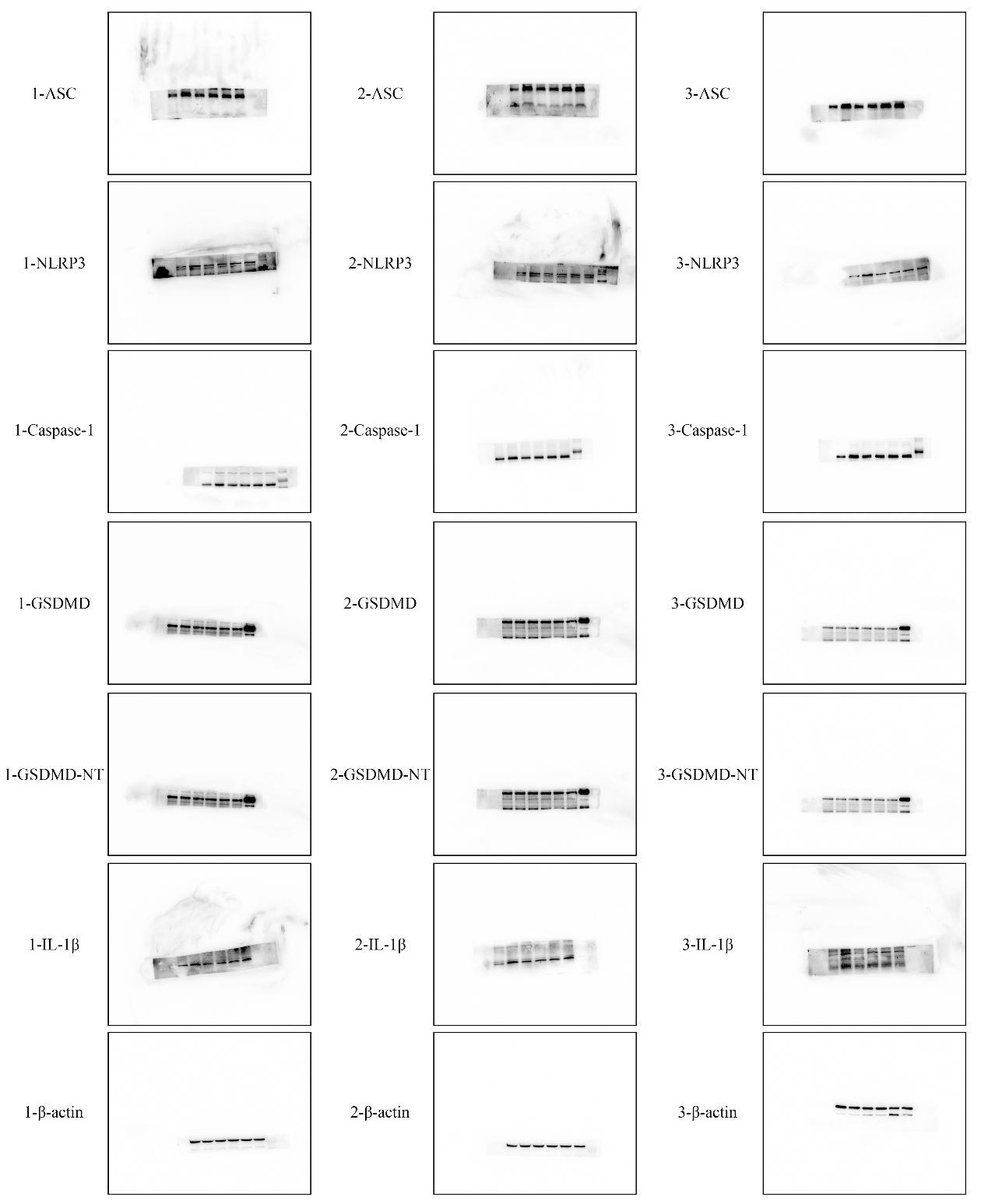

Supplement: Supplementary file 1 — Table S1: Liquid chromatography parameters. Table S2: Mass spectrometry conditions. Table S3: Primer sequences used for cell experiments. Table S4: Primer sequences used for rat experiments. Table S5: Identification of the chemical constituents of Rhodiola crenulata and Euonymus alatus extract by UHPLC‐Q Exactive HFX. Figure S1: Original western blot images for in vitro (cellular) experiments. Figure S2: Original western blot images for in vivo (animal) experiments. [file FSB2-40-e71824-s001.docx]
